# AZD5438 a GSK-3a/b and CDK inhibitor is antiapoptotic modulates mitochondrial activity and protects human neurons from mitochondrial toxins

**DOI:** 10.1038/s41598-023-35480-2

**Published:** 2023-05-23

**Authors:** Gongyu Shi, Helen Scott, Nur Izzah Farhana Mohamad Azhar, Andriana Gialeli, Benjamin Clennell, Keng Siang Lee, Jenny Hurcombe, Daniel Whitcomb, Richard Coward, Liang-Fong Wong, Oscar Cordero-Llana, James B. Uney

**Affiliations:** 1 Bristol Medical School, Translational Health Sciences, Dorothy Hodgkin Building, University of Bristol, Bristol, UK; 2 Bristol Renal, Dorothy Hodgkin Building, University of Bristol, Bristol, BS1 3NY UK

**Keywords:** Cell death in the nervous system, Diseases of the nervous system, Neuroscience

## Abstract

We previously reported that kenpaullone, which inhibits GSK-3a/b and CDKs inhibited CCCP mediated mitochondrial depolarisation and augments the mitochondrial network. To investigate the actions of this class of drug further, we compared the ability of kenpaullone, alsterpaullone, 1-azakenapaullone, AZD5438, AT7519 (CDK and GSK-3a/b inhibitors) and dexpramipexole and olesoxime (mitochondrial permeability transition pore inhibitors) to prevent CCCP mediated mitochondrial depolarisation and found that AZD5438 and AT7519, were the most effective. Furthermore, treatment with AZD5438 alone increased the complexity of the mitochondrial network. We also found that AZD5438 prevented the rotenone induced decrease in PGC-1alpha and TOM20 levels and that it mediated powerful anti-apoptotic effects and promoted glycolytic respiration. Importantly, experiments in human iPSC derived cortical and midbrain neurons showed AZD5438 mediated significant protective effects, preventing the neuronal cell death, and collapse in the neurite and mitochondrial network associated with rotenone treatment. These results suggest drugs that target GSK-3a/b and CDKs should be developed and assessed further as they may have significant therapeutic potential.

## Introduction

Mitochondrial dysfunction is associated with neurodegenerative disorders such as, Parkinson’s disease (PD), Alzheimer’s disease (AD), Huntington’s chorea and amyotrophic lateral sclerosis (ALS). Mitochondria generate ATP by oxidative phosphorylation and provide most of the energy for cellular processes. In addition, mitochondria play a direct role in controlling cellular calcium levels, are pivotal to antioxidant pathways and regulate lipid and amino acid metabolism and apoptosis^1–3^. Mitochondria undergo continuous rounds of fusion, fission, removal, and biogenesis to ensure the cell has a population capable of meeting its metabolic demands. Recent studies show that both glycolysis and oxidative phosphorylation provide neurons with energy during periods of particularly high demand^4,5^. Therefore, a deterioration in the glycolytic process and/or mitochondrial function will adversely alter neuronal processes such as synapse, formation, vesicle transport and recycling and protein synthesis, processing, and degradation. Ageing is also associated with decreases in both glycolysis and mitochondrial function^6,7^. Interestingly, synaptic mitochondria have been found to have a lower membrane potential than mitochondria proximal to the soma, and there is increased risk of mitochondria dysfunction at these distal dendritic sites^8,9^.

We have previously used a high-content assay of parkin recruitment to identify small molecules that modulate mitochondrial function. Our screen of a neuroactive compound library identified Kenpaullone (an inhibitor of GSK3α/β and CDKs) as the most significant non-toxic modulator of parkin recruitment^10^. We also found that kenpaullone augmented the mitochondrial network and reversed the CCCP-induced perinuclear clustering and fragmentation of mitochondria, thereby maintaining a healthier network throughout the cytoplasm^10^. Additionally, kenpaullone was shown to reverse the loss of mitochondrial membrane potential (Ψm) and toxicity caused by treatment of cells with the complex I inhibitor MPP+. These observations suggest that drugs that target GSK3α/β and CDKs could be developed to treat neurodegenerative conditions. We therefore compared compounds that: acted in a similar manner to kenpaullone (i.e., inhibited CDKs and GSK-3), had better pharmacodynamic properties and were being used in preclinical/clinical studies. Kenpaullone has undergone preclinical development for amyotrophic lateral sclerosis (ALS)^11^ and its derivatives alsterpaullone and 1-azakenapaullone protected podocytes by preventing apoptosis following activation of GSK3α/β and p38 mitogen-activated protein kinase pathways^12^. A small molecule screen to identify drugs for the treatment of ALS identified kenpaullone as its top hit^11^. GSK3 inhibitors target the mitochondrial membrane transition pore and this study showed that kenpaullone mediated superior neuroprotective effects than olesoxime and dexpramipexole, drugs that target the mitochondria permeability transition pore (mPTP) but do not inhibit GSK-3 or CDKs. Kenpaullone was found to have the potential to treat ototoxicity, and to find drugs that acted in a similar manner a kinase screen was conducted and AZD5438 and AT7519 identified^13^. AZD5438 and AT7519 were found to inhibit GSK3α/β and CDKs with high potency, protect cells from toxin induced damage and have more favourable drug absorption, distribution, and penetrance properties than kenpaullone^13,14^. We therefore assessed the therapeutic potential of alsterpaullone, 1-azakenapaullone, AZD5438, AT7519, and for comparison dexpramipexole (KNS-760704) and olesoxime (TR019622) using assays to measure, mitochondrial morphology, apoptosis, mitochondrial DNA (mtDNA) content and neuroprotection assays.

## Results

### The CDK and GSK3 beta inhibitors AZD5438 and kenpaullone prevent CCCP induced changes in mitochondrial morphology and potential

**Figure 1:**
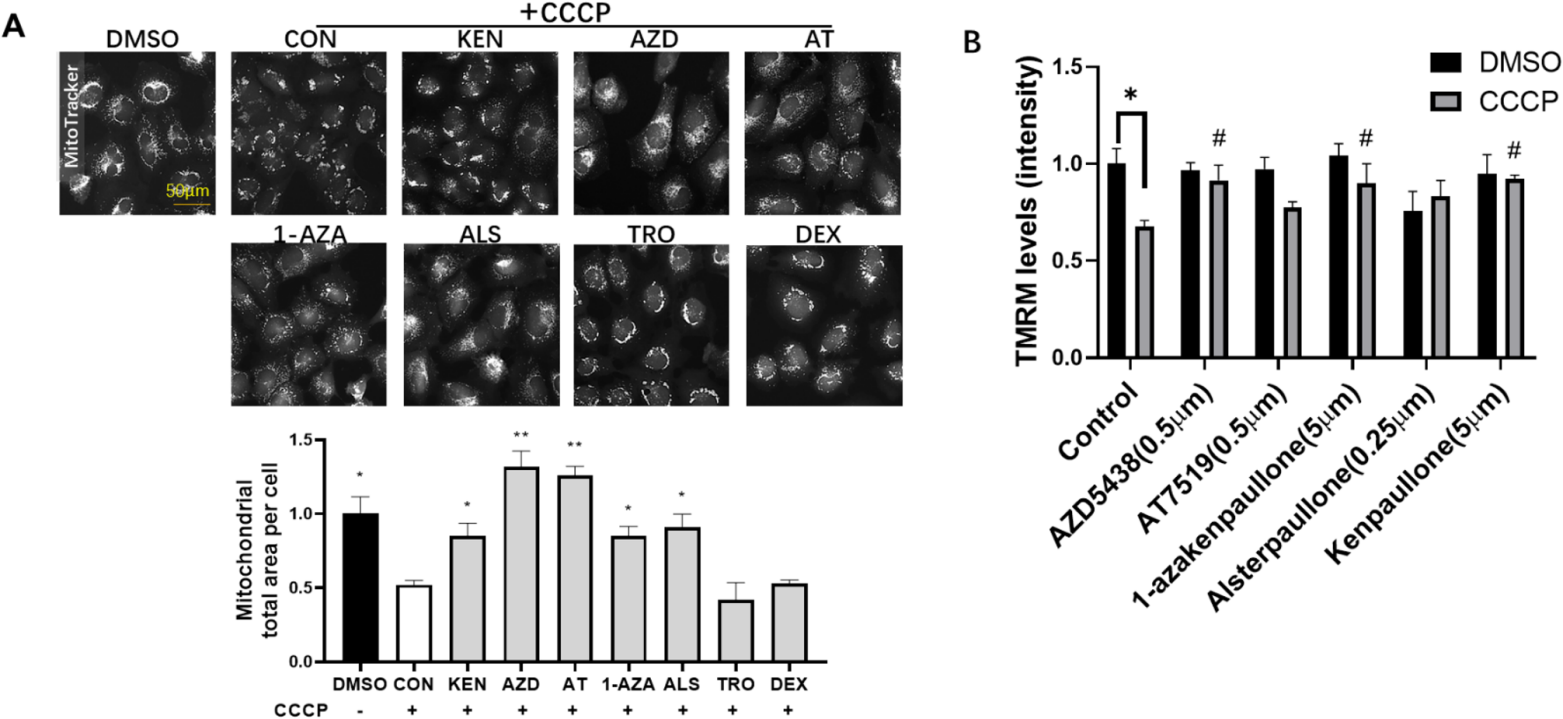
The effect of GSK/CDK inhibitors on mitochondrial area and membrane potential. (**A**) Representative images of mitochondria stained with MitoTracker Red following treatment with the indicated GSK/CDK inhibitors prior to incubation with CCCP. The values from each group were normalised to controls. The bar graphs show total mitochondrial area per cell (means + SEM of 3 independent experiments) obtained using the automated INCell Analyser capture protocol. Following treatment with CCCP several of the compounds prevented the decrease in mitochondrial area seen following treatment with CCCP when compared to the plus CCCP control. Statistical analysis was carried out by one-way ANOVA test with Sidak's multiple comparisons. *p < 0.05, **p < 0.01, ***p < 0.001. (**B**) H4 cells were treated with GSK/CDK inhibitors for 24 h prior to incubation with CCCP (10uM for 2 h) and TMRM levels measured. Statistical analysis was carried out by two-way ANOVA test with Tukey's multiple comparisons test. Mean ± SEM of 3 independent experiments are shown. *p < 0.05. Asterisks denote comparisons between negative (DMSO) and positive (CCCP) control, hashes denote comparisons between positive control and GSK/CDK inhibitors treatments (plus CCCP).

We previously reported that kenpaullone, an inhibitor of GSK-3a/b and cyclin dependent kinases (CDK’s), inhibited parkin recruitment to mitochondria and protected cells from MPP+ a mitochondrial complex 1 inhibitor toxin ^10^. To investigate these findings further we selected drugs that inhibited CDKs and GSK3α/β (AZD5438, alsterpaullone, 1-azakenapaullone and AT7519) and for comparison those that target the mPTP (olesoxime or dexpramipexole) and investigated their ability to modulate mitochondrial activity and protect neuronal cells from injury. We first compared the ability of these drugs to prevent the changes in mitochondrial morphology associated with treatment with a depolarising agent. To achieve this, H4 neuroglioma were treated with carbonyl cyanide 3-chlorophenylhydrazone (CCCP) to disrupt the mitochondrial membrane potential (Ψm), and high-magnification images captured and analysed using the In Cell analyser^10^. We also monitored cell number using the InCell Analyser and no significant decrease in cell viability was seen with the drug treatments (Fig. S1A). We quantified CCCP-induced mitochondrial perinuclear clustering and fragmentation using the InCell Workstation measures “organelle—total area” and “organelle—elongation”. We found that AZD5438 and its sister compound AT7519 were the most effective at preventing changes in mitochondrial morphology followed by the paullone compounds, however, neither olesoxime nor dexpramipexole significantly reversed the reduction in mitochondrial area caused by CCCP (Fig. 1A). It should be noted that dose response curves for each inhibitor were conducted prior to these assays to identify the effective non-toxic dose range. We also used H4 neuroglioma cells stably expressing parkin-EGFP to compare recruitment of parkin following CCCP to benchmark the drugs against kenpaullone (Fig. S1B). We found that only treatment with AZD5438 and kenpaullone mediated a significant inhibitory effect on parkin recruitment (Fig. S1B,C). High magnification images show examples of the clustering and formation of parkin puncta (measured by the In Cell analyser in Fig. S1C). The addition of AZD5438 can also be seen to prevent this clustering and a normal distribution and expression of parkin is maintained. (Fig. S1C). We also visualised mitochondria using the InCell analyser to assess the mitochondrial form/elongation factor, which is a measure of spherical and fragmented mitochondria^10^. Experiments showed that AZD5438, AT7519 and 1-azakenapaullone protected cells from mitochondrial fragmentation (Fig. S1D). We further assessed mitochondrial membrane potential using the tetramethyl rhodamine methyl ester (TMRM) dye and found that AZD5438, kenpaullone and 1-azakenapaullone significantly inhibited the CCCP induced loss of membrane potential (Fig. 1B).

### AZ5438 mediates significant protective effects against mitochondrial complex 1 inhibitors MPP+ and rotenone

**Figure 2:**
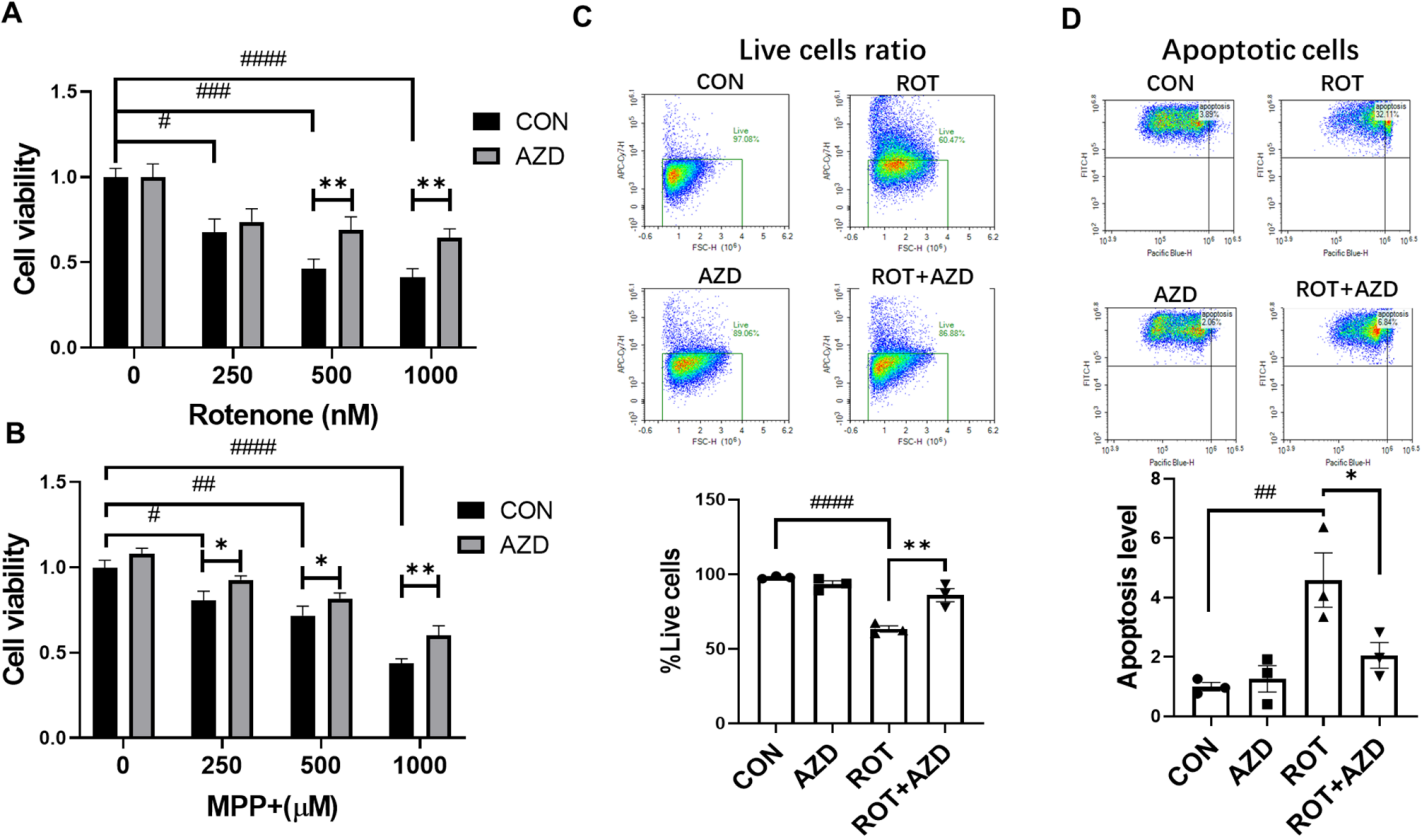
Assessing the protective effects of AZD5438 using cellular models of Parkinson’s disease. Cells were incubated with various concentrations of rotenone (**A**) and MPP+ (**B**) and the cytotoxic effects assessed using MTT assays. Cells also treated with AZD5438 (250 nM) were protected from the toxic effects of both rotenone and MPP^+^. Flow cytometry using a live/dead cell indicator dye was used to assess the percentage of live cells in the total population in each treatment (**C**) and apoptotic cell (**D**) ratios. Gating was carried out on forward and side scatter properties. Heat maps show the square gate sorting live cells based on low live/dead cell dye intensity. The quadrant identifies apoptotic cells in the live cell population based on high intensity Hoechst staining. The bar graphs show the percentage of live cells and apoptotic cell levels following treatment with rotenone (500 nM) and AZD5438 (250 nM). Statistical analysis was carried out by two-way ANOVA test with Tukey's multiple comparisons test. Means ± SEM of 3 independent experiments are shown. * and ^#^ = p < 0.05, ** and ^##^ = p < 0.01, *** and ^###^ p < 0.001, **** and ^####^ = p < 0.0001.

AZD5438 was highly effective at inhibiting the loss in mitochondrial membrane potential and in mitochondrial fragmentation and we therefore investigated whether this CDK and GSK inhibitor, could mediate protective effects against the mitochondrial complex-1 inhibitors, MPP^+^ and rotenone^10^. Exposure to MPP+ and the insecticide rotenone have been used extensively to mediate mitochondrial toxicity and model neurodegenerative conditions^15–17^. Our results showed that AZD5438 protected H4 cells (as assessed by MTT assays) against the cytotoxic effects of both rotenone and MPP^+^ (Figs. 2A,B and S2). Further, analysis using Mitotracker to stain for mitochondria showed treatment with rotenone decreased the cell count and mitochondrial area and mediated the fragmentation of mitochondria and that AZD5438 reversed these effects (Fig. S2). In addition, treatment with AZD5438 alone was found to robustly increase total mitochondrial area and significantly decrease the elongation factor indicating more elongated mitochondria (Fig. S2). High magnification images (Fig. S2A) mirror the results obtained in Fig. S1D and Fig. 2D) showing that treatment with AZD5438 maintains the elongated mitochondrial form seen in DMSO treated controls as opposed to the fragmented rounded morphology seen in rotenone treated cells.

### AZD5438 protects cells from rotenone induced toxicity by inhibiting apoptosis and free radical production and preventing the downregulation of TOM20 and PGC-1 alpha

**Figure 3:**
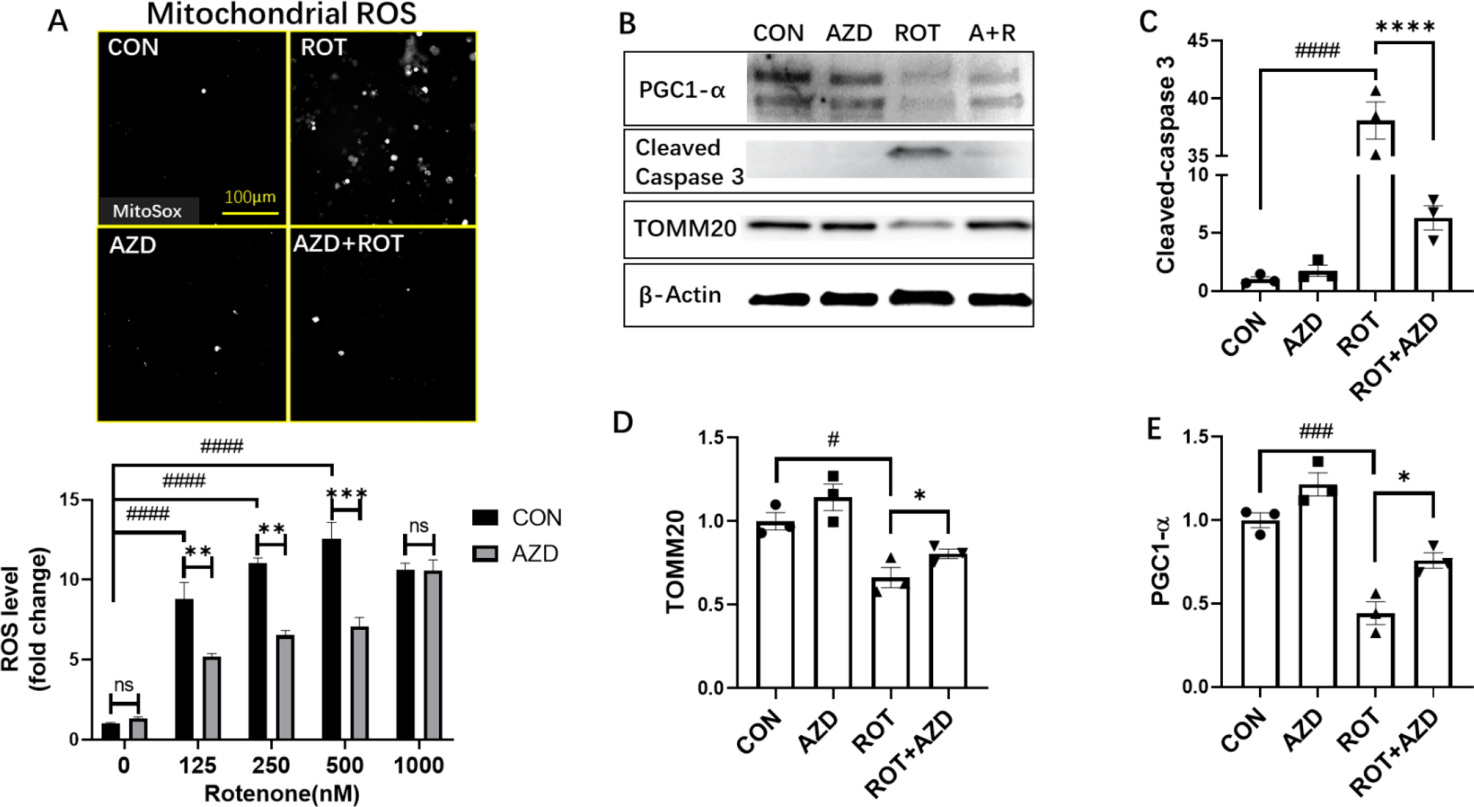
Evaluating the action of AZD5438 using assays of free radical production, apoptosis, and mitochondrial function. Representative photomicrographs take with the InCell Analyser 2200 of H4 cells stained with MitoSOX Red a mitochondrial superoxide indicator (**A**). Values in the bar graphs are the means ± SEM of 3 independent experiments. Representative western blots of PGC1-alpha, caspase 3 and TOMM20 following treatment with rotenone and AZD5438 are shown are shown (**B**). The bar graphs in C, D and E show the mean ± SEM of 3 independent experiment’s assessing the expression of cleaved caspase 3, TOMM20 and PGC1-alpha. Statistical analysis was carried out by two-way ANOVA with Tukey's multiple comparisons test. *p < 0.05, ***p < 0.001, ****p < 0.001, ****p < 0.0001.

Rotenone has been reported to induce apoptosis and to increase the production of mitochondrial reactive oxygen species^15^ and we therefore evaluated the action of AZD5438 on H4 cells using assays of apoptosis, free radical production, and mitochondrial function. Flow cytometry to sort cells stained with the dead cell permeant nuclear dye Zombie NIR™ and Hoechst showed that AZD5438 increased the live cell ratio and inhibited apoptosis following treatment with rotenone (Fig. 2C,D). AZD5438 also protected cells by significantly inhibiting the effects of rotenone on cell viability and the production of reactive oxygen species (ROS) (Fig. 3). We also used western blot analysis to investigate whether AZD5438 inhibited the cleavage of caspase 3 and assessed the effect of AZD5438 on the expression of peroxisome proliferator-activated receptor (PGC-1 alpha) and TOM20 (Fig. 3B–E and the unprocessed full blots are shown in Fig. S3). The results showed that AZD5438 significantly inhibited the rotenone mediated production of cleaved caspase-3 (Fig. 3B,C). PGC-1 alpha is a master regulator of mitochondrial respiration and biogenesis^16^, while TOM20 (translocase of the outer membrane) is bound to the outer mitochondrial membrane and is needed to regulate the import of proteins into mitochondria. Our results showed that treatment with rotenone down-regulated the expression of both TOM20 and PGC-1 alpha, while AZD5438 significantly inhibited this effect (Fig. 3D,E). Together these results indicate AZD5438 preserves mitochondrial function and protects cells from the mitotoxic actions of rotenone. The ability of AZD5438 to protect mitochondrial function was also investigated in SH-SY5Y neuroblastoma cell. Similar results to those obtained with H4 cells were observed and AZD5438 protected SHSY-5Y cells from the cytotoxic effects of rotenone and treatment with AZD5438 alone significantly increased cellular mitochondrial DNA content (Fig. S4).

### AZD5438 increases basal glycolysis and acts on CDK9 and CDK5 to modulate parkin recruitment

**Figure 4:**
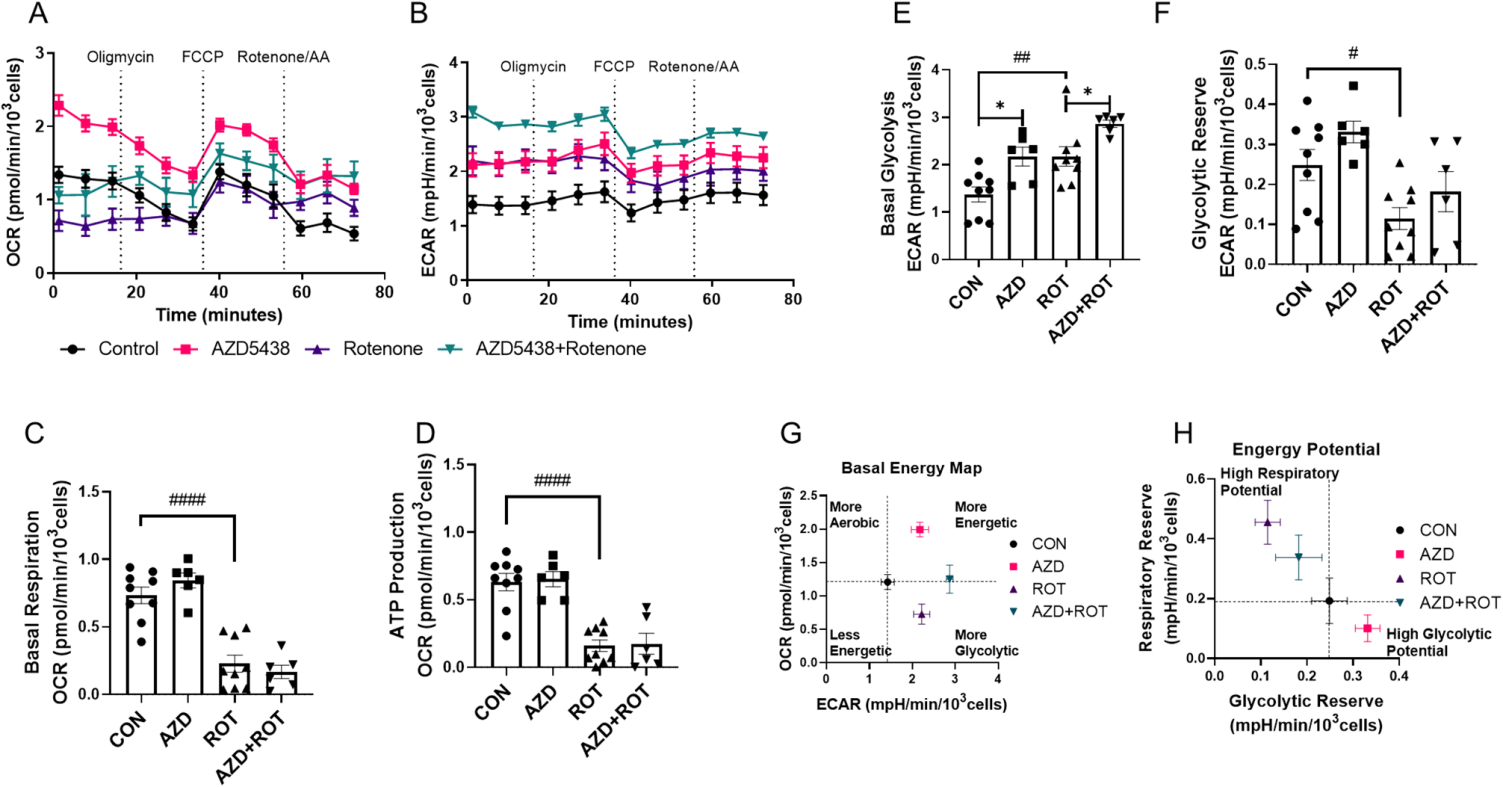
Assessing the effect of AZD5438 on cellular energy metabolism using the Seahorse analyser. (**A**) Oxygen consumption rate (OCR) over time is shown (C-D are based on these data). (**B**) Extracellular acidification rate (ECAR) over time (G-H are based on these data). (**C**) ROT significantly (p < 0.0001) reduced the mitochondrial basal respiration rate compared to CON. (**D**) ROT significantly (p < 0.0001) reduced the cellular ATP production rate compared to CON. (**E**) AZD significantly (p = 0.01) increased the cellular basal glycolysis rate compared to CON, as did ROT (p = 0.005) and AZD + ROT (p = 0.03) compared to ROT alone. (**F**) ROT significantly (p = 0.02) decreased the cellular glycolytic reserve. (**G**) The basal energy map plots OCR with ECAR at the basal level and shows AZD was more energetic, and ROT was more glycolytic (both compared to CON), while AZD + ROT was more energetic compared to ROT. (**H**) The energy potential map shows AZD had a higher glycolytic potential and ROT had the higher respiratory potential (both compared to CON), while AZD + ROT had higher glycolytic potential compared to ROT. Statistical analysis was carried out by two-way ANOVA test with Tukey's multiple comparisons test. Data from 3–4 biological replicates; data is displayed as mean ± SEM; significance is displayed as *p < 0.05, **p < 0.01, ***p < 0.001, ****p < 0.0001. Asterisks denote comparisons between AZD and CON or AZD + ROT with ROT, hashes denote comparisons between CON and ROT.

To further investigate the action of AZD5438 we used the Sea Horse Mito stress test protocol to monitor glycolytic and oxidative metabolism. This involves the sequential injection of drugs that disrupt the specific electron transport chain (ETC) complexes and allows oxygen consumption rate (OCR, Fig. 4A) and extracellular acidification rate (ECAR, Fig. 4B) to be recorded under conditions that alter mitochondrial respiration (described further in the “Seahorse experiments” section of Experimental procedures). Analysis of the OCR data (Fig. 4A) showed that rotenone treatment significantly reduced the mitochondrial basal respiration rate (Fig. 4C) and ATP production rate (Fig. 4D). While AZD5438 treatment did not reverse the action of rotenone on basal respiration or ATP production. The results further showed that treatment with AZD5438 had no effect on mitochondrial ATP production or basal respiration, however AZD5438 did mediate a significant increase in basal glycolysis (Fig. 4E). Treatment with rotenone alone significantly decreased glycolytic reserve and there was a trend towards rotenone increasing glycolysis Fig. 4F). When cells were treated with both rotenone and AZD5438 basal glycolysis was significantly greater than when treated with AZD5438 alone. The cells' basal energy map (OCR vs ECAR) at baseline before oligomycin injection (Fig. 4G) shows AZD5438 treatment renders cells more energetic (both more aerobic and more glycolytic), and rotenone treatment renders cells more glycolytic but less aerobic, while treatment with AZD5438 and rotenone made cells more energetic than when treated with rotenone alone. These results suggest AZD5438 upregulated the metabolic status of cells. The energy potential of the cells (Fig. 4H, respiratory reserve vs glycolytic reserve) shows that AZD5438 treatment upregulated the glycolytic potential of cells.

Inhibition of CDK-9 was recently reported to inhibit PINK1/PRKN mediated mitophagy via the regulation of sirtuin 1 (SIRT1)^18^ and AZD5438 has been reported to be a potent inhibitor of CDKs 1,2 & 9 and GSK-3α/β^13,19^. We therefore investigated whether AZD5438 inhibited the decrease in mitochondrial area and parkin recruitment (mediated by CCCP) via an action on CDK’s and/or via the inhibition of GSK-3β. To achieve this, we used siRNAs to knockdown the expression of CDK’s 1, 2, 5 and 9 and GSK-3β. The data showed that cells must be treated with the anti-CDK-9 siRNA for an effect on mitochondrial area and parkin recruitment to be seen (Fig. S5) and that AZD5438 did not mediate an effect on parkin recruitment in the presence of CDK5 and CDK9. These results suggest AZD5438 may act via CDK5 and 9 to inhibit parkin recruitment and that it exerts a partial action on the preservation of mitochondrial area via CDK9. However, treatment with an siRNA against GSK-3β (and investigation using a GSK-3β conditional knockout line^20^) had no effect on parkin recruitment (Fig. S5). SIRT1 has been shown to regulate brain energy metabolism^21^, protect against mitochondrial injury by inhibiting excess PINK1/Parkin activation^18,22^ and to inhibit GSK-3 activation^23^. We therefore investigated the action of AZD5438 and for comparison a selective GSK-3 inhibitor (CHIR99021) on SIRT1 activity and cell viability as assessed by MTT assays. We found that AZD5438 did not alter basal SIRT1 activity, however treatment of cells with CHIR99021 mediated a significant decrease in SIRT1 activity (Fig. S6A,B). We also found that cells treated with rotenone had significantly reduced SIRT1 activity and that treatment with both AZD5438 and CHIR99021 significantly inhibited this rotenone mediated decrease. The inhibition of basal SIRT1 levels by CHIR99021 (but not AZD5438) suggests AZD5438 is compensating for the GSK-3 mediated effect on SIRT1 via an action on CDK’s. However, although both AZD5438 and CHIR99021 significantly inhibit the rotenone mediated decrease in SIRT1 levels only AZD5438 was found to mediate a significant protective effect as assessed by MTT activity (Fig. S6C,D). These results suggest that the differential inhibitory actions of AZD5438 on GSK-3 and CDK mediates a more powerful protective effect that that seen by the inhibition of GSK-3 alone.

### AZD5438 protects primary rat neurones and human IPSC derived neurons from rotenone induced toxicity

**Figure 5:**
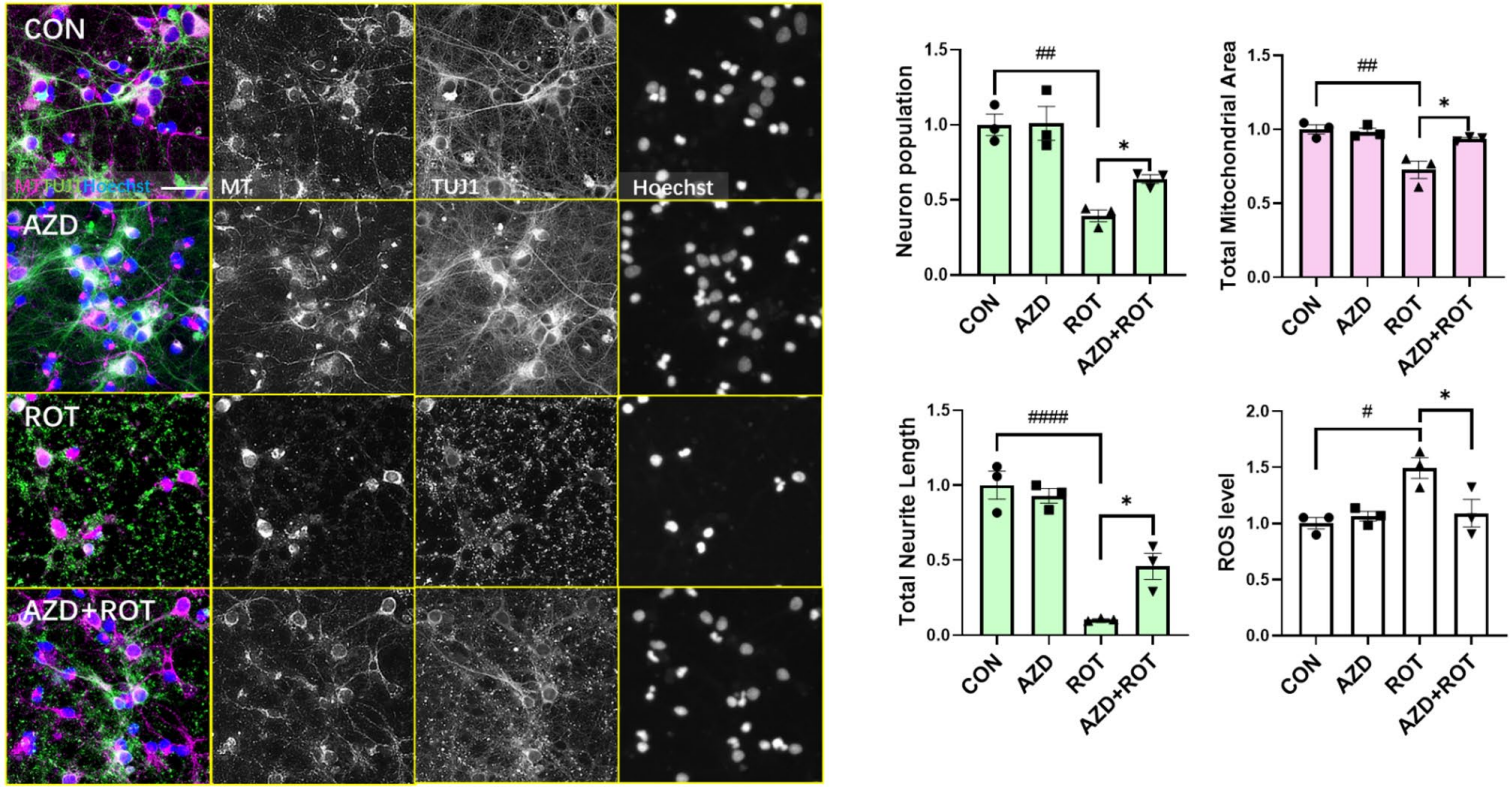
AZD5438 protects primary rat cortical neurons from rotenone induced reductions in neuronal and mitochondrial complexity. Representative photomicrographs of E18 primary cortical cultures treated with ROT and AZD for 24 h. Merged images are of neurons stained with Tuj1(green), Hoechst (blue nuclei) and MitoTracker, (Red mitochondria). Images were taken with the INCell Analyzer 2200 high-content imaging system. Individual grey scale channels showing the Mito Tracker-Red, Tuj1 and Hoechst following treatment with rotenone and AZD5438 are also shown. The bar graphs show the means ± SEM of 3 independent experiments and show that rotenone significantly reduced the neuronal cell population, neurite length, total mitochondrial area and increased ROS levels, while treatment with AZD5438 protecting against these effects. Total area represents all mitochondria in the experimental field. Statistical analysis was carried out by two-way ANOVA with Tukey's multiple comparisons test. *p < 0.05, **p < 0.01, ***p < 0.001, ****p < 0.0001.

We also investigated the action of AZD5438 on rat primary cortical neurons. Cells were incubated with rotenone for 24 h and labelled with MitoTracker, neuron-specific Class III β-tubulin (TUJ1, which labels neurons) and Hoechst and the protective effect of AZD5438 assessed using the InCell Analyser 2200 (Fig. 5). Following treatment with rotenone it was evident that the neurite network had become very fragmented (Fig. 5) and there was a significant loss of neurites as assessed by total neurite length (Fig. 5). There was also a significant loss of neuronal cell bodies following treatment with rotenone (Fig. 5 neuronal population measure). A loss of mitochondrial staining also coincided with the loss in neurites, however MitoTracker staining-intensity in the surviving neuronal cell bodies appeared to be increased though there was an overall significant loss in mitochondria (Fig. 5). This increase in cell body MitoTracker staining may be due the few surviving neurons needing to mediate a compensatory increase in mitochondrial fission. As well as mediating a significant reduction in the cell population, neurite length and mitochondrial area, treatment with rotenone also mediated an increase in neuronal ROS levels. Treatment with AZD5438 alone did not cause a change in any of the parameters measured when compared to controls. However, treatment with AZD5438 inhibited the inhibited the production of ROS and prevented the loss of nerites, cell bodies and mitochondria mediated by rotenone (Fig. 5).

**Figure 6:**
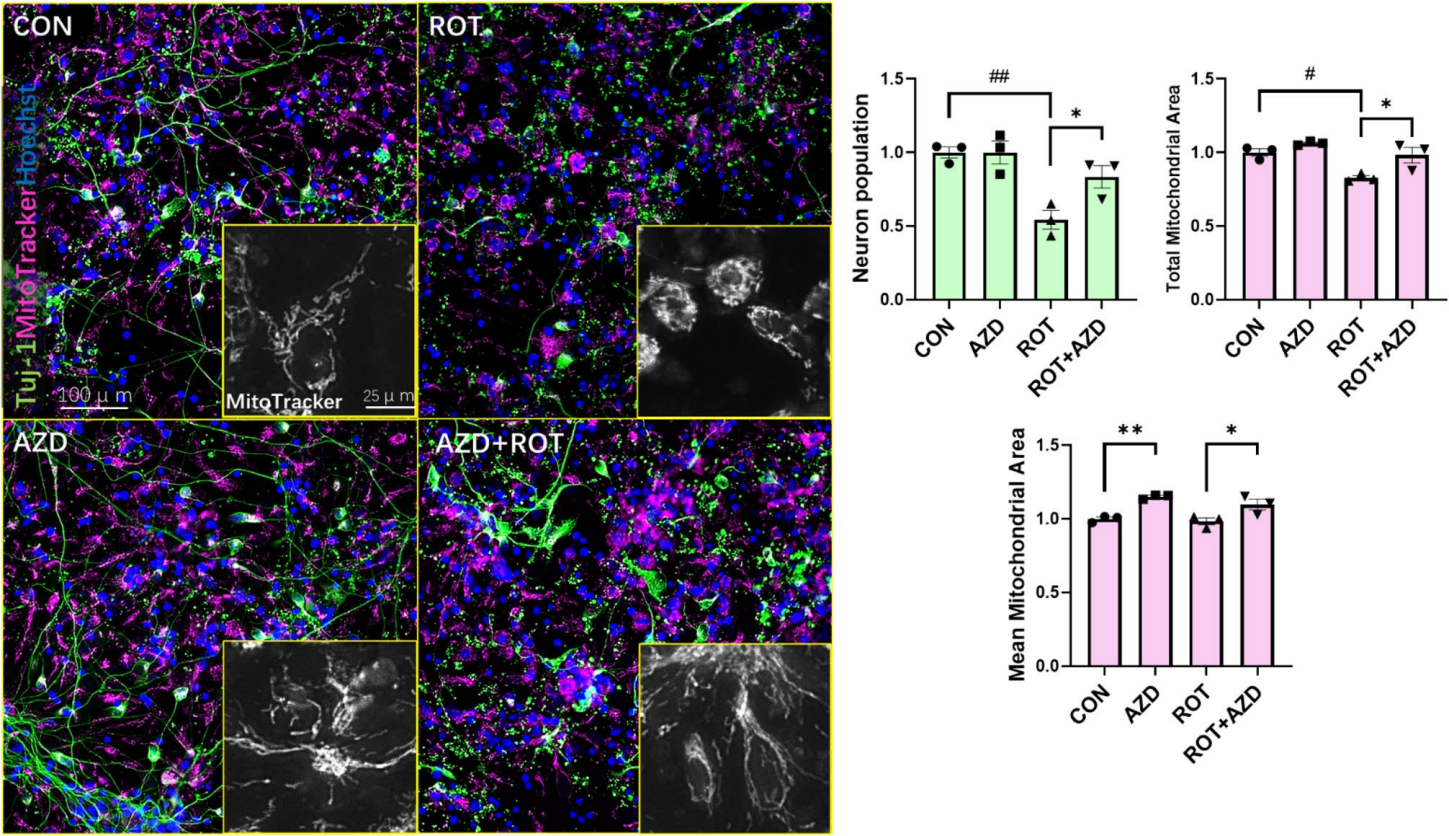
AZD5438 protects human midbrain neurons from rotenone induced reductions in neuronal and mitochondrial complexity. Representative photomicrographs of midbrain cultures derived from human iPSC treated with ROT and AZD for 24 h. Merged images are of neurons stained with Tuj1(green), Hoechst (blue nuclei) and MitoTracker, (Red mitochondria). Inset grayscale images are of mitochondria stained with MitoTracker-Red taken with the INCell Analyzer 2200 high-content imaging system. The bar graphs show the means ± SEM of 3 independent experiments and show that rotenone significantly reduced the neuronal cell population, mean and mitochondrial area, while AZD5438 protected against the rotenone mediated toxicity. Statistical analysis was carried out by two-way ANOVA with Tukey's multiple comparisons test. *p < 0.05, **p < 0.01, ***p < 0.001.

**Figure 7:**
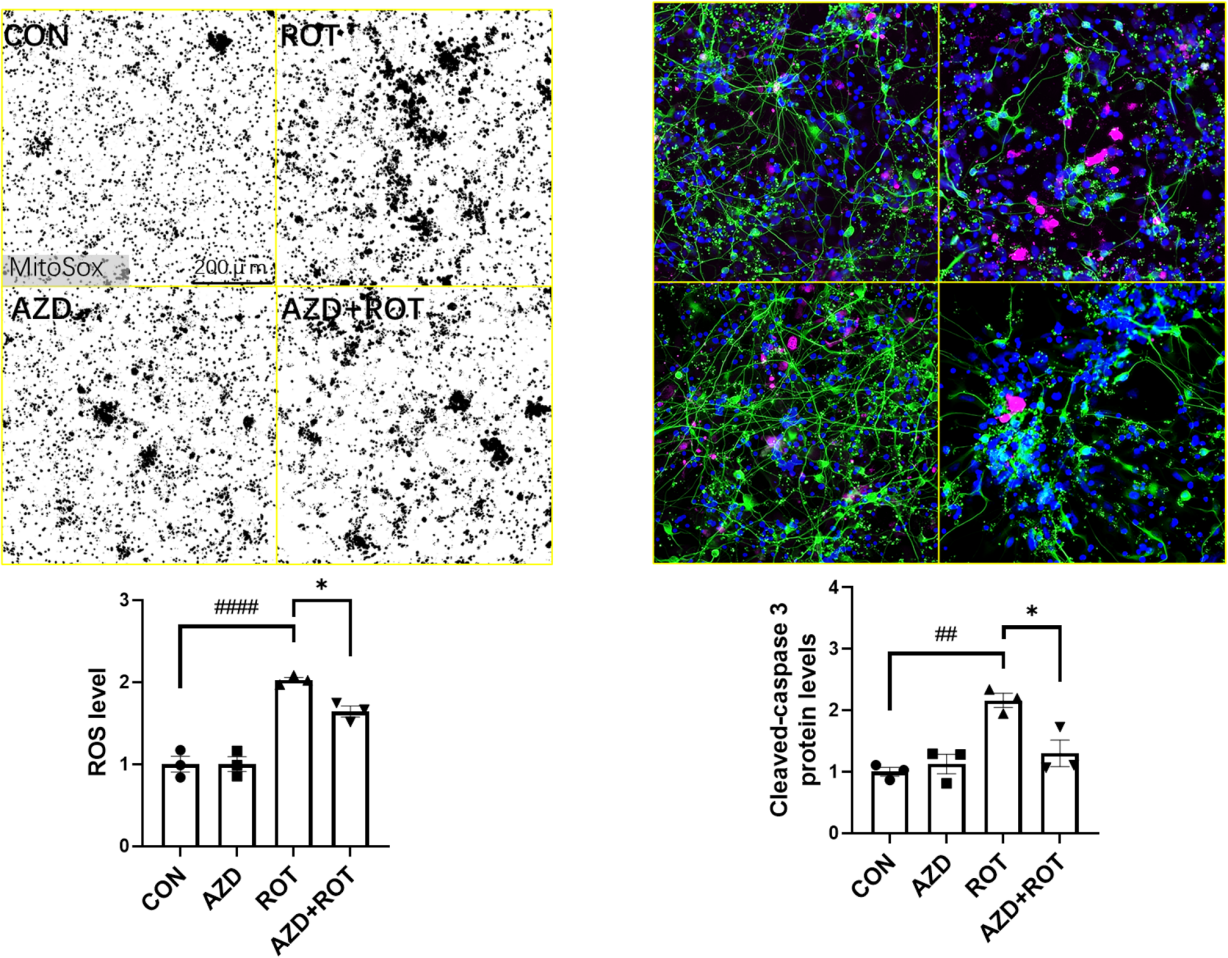
AZD5438 protects human midbrain neurons from rotenone induced free radical production and apoptosis. Midbrain cultures derived from human iPSC were treated with ROT and cells stained with MitoSox and immunocytochemically labelled with C-Cas3 Ab to allow imaging and quantification of reactive oxygen species and cleaved caspase 3 respectively. The bar graphs show the means ± SEM of 3 independent experiments. Statistical analysis was carried out by two-way ANOVA with Tukey's multiple comparisons test. *p < 0.05.

We next investigated the neuroprotective effects of AZD5438 in human midbrain neurons (Figs. S6, 6 and 7) derived from induced pluripotent stem cells (iPSCs). We again found that treatment with rotenone reduced mitochondria number as assessed by measures of mitochondrial area (Fig. 6) and increased ROS production and apoptosis as assessed by cleaved caspase-3 (Fig. 7). Treatment with AZD5438 alone was found to increase the area of the mitochondrial network in these midbrain neurons. A result that supports our observation that AZD5438 increases mtDNA (Fig. S4). We also found that treatment with AZD5438 prevented the neuronal damage mediated by rotenone as seen by the survival of neuronal cells, decreased loss in total mitochondrial area and complexity (Fig. 6), prevention of ROS and caspase-3 production (Fig. 7) and MTT activity (Fig. S7). Interestingly, preliminary patch clamp analysis showed human iPSC derived neurons treated with AZD5438 were protected from the rotenone mediated decrease in neuronal membrane potential (Fig. S8). Together these data show AZD5438 allows neurons to maintain their physiological functions following acute exposure to toxins associated with mitochondrial damage.

## Discussion

We previously reported that kenpaullone, which inhibits GSK-3a/b and CDKs regulates mitochondrial function and protected cells from injury^10^. Iimportantly, kenpaullone, which inhibits GSK3α/β and CDKs was found to protect motor neurons and promote their survival more effectively than comparable inhibitors of GSK3 alone^11^. In addition, AZD5438 and AT7519 which inhibit GSK3α/β with a similar potency to kenpaullone but have different CDK inhibitory profiles, were found to be more effective inhibitors of cisplatin mediated toxicity and apoptosis than kenpaullone ^13,24^. We identified available GSK3α/β and CDK inhibitors and drugs that also targeted the mitochondrial permeability transition pore (mPTP) and compared their ability to modulate mitochondrial function with that of kenpaullone. We found that treatment with AZD5438 prevented the disruption of the mitochondrial membrane potential following treatment with CCCP and fragmentation of the mitochondrial network associated with CCCP treatment. Furthermore, treatment with AZD5438 alone increased the complexity of the mitochondrial network. AT7519, kenpaullone and its analogues, alsterpaullone and 1-azakenapaullone also inhibited CCCP mediated parkin recruitment and mitochondrial fragmentation though not as effectively as AZD5438. As these drugs have similar inhibitory action of GSK3a/b but differ in their anti-CDK profiles their differential action on parkin recruitment and the mitochondrial network must at least in part be governed via an action on CDKs. Dexpramipexole (KNS-760704) and olesoxime (TR019622), compounds reported to target the mPTP ^25^ and assessed in phase II ALS trials ^26^, had no effect on parkin recruitment or mitochondrial form. Kenpaullone which as a GSK3 inhibitor also inhibits the formation of the mPTP^27^ was previously found to protect motor neurons more effectively than Dexpramipexole (KNS-760704) and olesoxime (TR019622)^11^. Hence, our findings support the suggestion that drugs that target CDKs and GSK3 are powerful protectors of mitochondrial function.

Kenpaullone protects cells from the mitochondrial respiratory chain complex I inhibitors^10^ and we found that AZD5438 also mediated significant protection against the complex 1 inhibitors rotenone and MPP+. We evaluated the mechanism by which mitochondria were protected and found that AZD5438 increased cellular levels of mitochondrial DNA, promoted glycolytic respiration, and exerted powerful anti-apoptotic effects. We also found that AZD5438 prevented the rotenone induced decrease in PGC-1alpha and TOM20 levels, again indicating the preservation of mitochondrial function. We also confirmed a previous finding^18^ that showed CDK9 (and possibly CDK5) inhibitors prevent CCCP induced parkin recruitment. These experiments also suggested that CDK9 may be involved in regulating mitochondrial form. Importantly, we wished to assess the reproducibility and relevance of our findings to human neuronal physiology and therefore evaluated AZD5438 in human iPSC derived neurons. We found that AZD5438 mediated significant protective effects, preventing the ROS production, neuronal cell death, and collapse in the neurite and mitochondrial network associated with rotenone treatment. In vitro models using primary rodent and human neurons confirm that rotenone disrupts mitochondrial complex I, leading to free radical production and the induction of apoptosis in part by induction of the mPTP ^15,28,29^. In addition, a recent RNA-seq study of 215 iPSC lines found that those differentiated to midbrain neurones were uniquely sensitive to rotenone toxicity^28^. A recent study showed that disrupting the function of mitochondrial complex 1 was sufficient to cause human-like parkinsonism in mice^30^. Together these results strongly suggest CDK and GSK-3 inhibitors that protect mitochondrial function may have potential as anti-parkinsonian therapeutics.

Previous studies have shown that inhibition of either CDK or GSK-3 pathways mediate actions on mitochondrial quality control, parkin mediated mitophagy, apoptosis and cell survival^18,31,32^. For instance, several CDK’s have been shown to be neuroprotective; CDK5 which regulates glucose metabolism, endocytosis and neurite growth has been implicated in the aetiology of PD and Alzheimer’s disease (AD)^32,33^. While the multi-kinase inhibitor, G209A, was shown to restore function in an A53T-α-synuclein model of PD^34^. Significantly, the inhibition of CDK2 by AZD5438 (and kenpaullone) has previously been shown to protect cells from cisplatin induced mitochondrial ROS production and apoptosis^13,24^. GSK3β inhibitors mediate their protective effects via several pathways, for instance GSK3β has been shown to regulate hexokinase 2 mediated mitochondrial toxicity/protection^27^ and GSK3β activity increases mPTP and Bax mediated apoptosis. In preclinical studies GSK inhibitors have proven to be effective at inhibiting the onset of neurodegenerative illnesses in models of AD, ALS and PD (reviewed in^27^. GSK3α/β inhibitors also regulate mitochondrial quality control and increase mitochondrial biogenesis^35^ and this may occur via the regulation of PGC1α and Nrf2 which are key regulators of mitochondrial respiration and biogenesis^35^. For example, the inhibition of GSK3β was shown to upregulate PGC1α and increase mitochondrial biogenesis and activity^27^. These data suggest that the AZD5438 mediated increase in mitochondrial DNA could occur due to the inhibition of GSK3β regulated pathways. However, inhibition of CDK5 has been shown to upregulate the expression of peroxisome proliferator-activated receptors-gamma (PPARγ)^36^ which regulates PGC1α, mitochondrial biogenesis and glucose metabolism^37^. These data therefore suggest that the AZD5438 mediated increase in mitochondrial DNA could occur due to the inhibition of CDKs mediating a slowing of parkin mediated mitophagy and/or due to an action on PGC1α. Also of significance are the observations that: (i) CDKs regulates SIRT1 expression^18^; (ii) SIRT1 regulates energy metabolism^21^; (iii) SIRT1 expression inhibits PINK1/Parkin activation and excess mitophagy^18,22^ and thereby protects cells against mitochondrial injury^18,22^; (iv) there is a reciprocal relationship between SIRT1 expression and GSK-3 expression^23^. We showed that both AZD5438 and CHIR99021 a specific GSK-3 inhibitor significantly increased SIRT1 expression following exposure to rotenone. However, only AZD5438 mediated a significant protective effect against rotenone toxicity and CHIR99021 decreased SIRT1 expression in untreated cells. Together, these data suggest that the inhibitory action of AZD5438 on both GSK3α/β and CDK pathways results in the activation of pro-survival pathways that are more effective and are distinct to those pathways activated by the inhibition of GSK3α/β alone.

Inhibition of CDK may increase glycolysis via the upregulation of PPARγ^36,37^ and studies have also shown that the inhibition of GSK-3 pathways increases glycolysis^27,37,38^. Interestingly, recent research shows that energy from glycolysis and not oxidative phosphorylation is needed to maintain basal synaptic transmission at the presynaptic terminals^5^ and that glycolysis outstrips oxidative metabolism in neurons to provide immediate energy supplies during periods of high energy demand^39^. In addition. mitochondria at distal dendritic sites have a lower membrane potential than mitochondria proximal to the soma, and distal mitochondrial produce free radicals and activate caspase-3 following a stress more readily than that those at the soma^8,9^. Significantly, enhancing glycolysis was found to slow or prevent neuronal cell loss in toxin and genetic animal models of PD and patient groups receiving drugs that enhanced glycolysis had a lower incidence of Parkinson’s disease^40^. These observations suggest that the decrease in glycolysis (^6,7,41^ that occurs with age would result in reduced/insufficient energy production during periods of high demand that compromise basal synaptic transmission. Dopaminergic neurons have exceptionally large synaptic networks and high energy requirements, and they would be particularly susceptible to this detrimental alteration in energy metabolism. In addition, a consequence of a decrease in glycolysis in neurons would first be to increase demand and stress on distal mitochondria. These findings, suggest that the anti-apoptotic, pro-glycolytic and mitochondrial membrane preserving actions of AZD548 may be beneficial to distal mitochondria, and acute treatment may delay the onset of the synaptic loss and neurodegeneration associated with aging and neurodegenerative disease. Chronic treatment with these drugs may be contraindicated due to their inhibitory action on the recruitment of parkin. However, it may be possible to design and/or screen for kinase inhibitors that do not modulate parkin recruitment but do promote glycolysis, are anti-apoptotic and maintain mitochondrial membrane potential and health. Collectively, this work suggests that drugs such as AZD5438 that inhibit both GSK3α/β and CDKs may confer protections to cells when they are under physiological stresses associated with the aetiology of neurodegenerative diseases such as PD and AD. Further investigation in animal models and subsequently small human trials will however be needed to assess the utility of such drugs.

## Experimental procedures

### Human cell lines culture

H4 cells (a gift from Takeda UK) were maintained in Dulbecco's modified Eagle's medium (Sigma–Aldrich, D6546) supplemented with 10% foetal bovine serum (Life Technologies, Inc., 10500064), 2 mm l-glutamine (Sigma–Aldrich, G7513), and 5000 units/ml penicillin/10 μg/ml streptomycin (Sigma–Aldrich, P4458). SH-SY5Y cells (ATCC, no. CRL-2266) were maintained in Dulbecco's modified Eagle's medium/F-12 (1:1) (Thermo Fisher Scientific, 21331020) supplemented with 10% foetal bovine serum (Life Technologies, 10500064), 2 mm l-glutamine (Sigma–Aldrich, G7513), and 5000 units/ml penicillin/10 μg/ml streptomycin (Sigma–Aldrich, P4458). Cells were passaged to new flasks when reaching 80% confluence in flasks. All cells were maintained in a humidified incubator at 37 °C, 5% CO_2_.

### Primary neuronal culture

Plates/coverslips were coated with 0.1 mg/mL Poly-D-Lysin (Gibco™, A3890401) one day prior to plating. E18 brains were dissected, and the cortex collected on ice-cold HBSS in dishes. Trituration of the tissue was performed using 1X Trypsin–EDTA (Sigma, T417) at 37 °C for 30 min and the cells seeded on plates and cultured in Neurobasal medium (Gibco 21103-049) supplemented with 1 × B27 (Gibco 17504-044), 2 mm l-glutamine and 5000 units/ml penicillin/10 μg/ml streptomycin. Medium was changed every four days/according to confluence until day 14.

We confirm that all animal experiments were carried out in accordance with the United Kingdom Animals Scientific Procedures Act (1986) and associated guidelines and were approved by the University of Bristol licence holder and ethics committee. The experiments were carried out and reported in accordance with ARRIVE guidelines.

### Human iPSC culture and differentiation towards midbrain neurons

The normal SNCA2 (NAS2) human induced pluripotent stem cells (hiPSC) line was a gift from Dr. Tilo Kunath, at the Centre for Regenerative Medicine, University of Edinburgh. The NAS2 lines were maintained and passaged as required to maintain morphology and cell number. Briefly, cells were seeded onto Vitronectin-coated plates containing complete Essential 8 (E8) feeder-free media at a density of 5 × 10^4^ cells/cm^2^ and incubated in a humidified incubator at 37 °C with 5% CO_2_. Cells were passaged at 75% confluency or when cells were seeded on to plate for 4–5 days. NAS2 hiPSCs derived midbrain dopaminergic neural culture were generated following our previously published protocol^42^. Briefly, healthy and functioning NAS2 hiPSCs were used for mDAN differentiation by first exchanging complete E8 media with neuron differentiation media 1 (NDM1). NDM1 contained N2B27 supplemented with neural fate inducing factors (100 nM LDN193189 and 10 μM SB431542) and patterning factors (400 ng/mL SHH C24II and 1.0 μM CHIR99021). On day 9, media was changed to N2B27 and on day 11, media was replaced by neuron differentiation media 2 (NDM2), which was supplemented with N2B27, neurotrophic factors (20 ng/mL BDNF, 20 ng/mL GDNF) and 0.2 mM ascorbic acid. Day 16, cells were passaged and plated onto polyornithine/Laminin coated coverslips or plates as required. Cells were maintained and maturated with neuron feeding media (NFM), at 20% O2, 5% CO_2_, 37 °C. Cells were passaged on day 3, 7, 12 and 16 and 10 μM ROCK inhibitor (Y-27632) was used in the media to enhance the survival of cells.

### Parkin recruitment and mitochondrial imaging assays

The Parkin recruitment assays were previously reported in detail^43^. In brief, cells expressing EGFP-Parkin were seeded in black-walled 96-well plates (Corning, 3904). and cells pretreated with the specified drugs for 24 h. When needed cells were transfected with siRNAs for 48 h and treated with CCCP (15 μm) for 2 h, followed by fixation and Hoechst staining. Plates were then imaged and quantified using the InCell Analyzer.

For quantitation of mitochondrial networks, MitoTracker Red CMXRos (Invitrogen, M7512) was used following manufacturer (Invitrogen) instructions. Mitochondrial membrane potential was measured using Tetramethylrhodamine (TMRM) (Invitrogen, I34361) with a working concentration of 30 nM in live cells at 37 °C and quantitated using the InCell analyser.

MitoSOX Red reagent (Invitrogen, M36008) was used to detect the superoxide ROS produced by mitochondria (Invitrogen). MitoSOX Red was used at a, 1:1000 dilution for 5 μM working solution in live cells for 15 min at 37 °C prior to imaging and analysis.

### Compounds

AT7519 (APE, A5719), TRO 19,622 (Bio-Techne, 2906), AZD5438 (Bio-Techne, 3968), dexpramipexole (Sigma, SML0392), 1-azakenpaullone (Sigma, A3734), alsterpaullone (Sigma, A4847) and kenpaullone (Sigma, K3888) were purchased and dissolved in DMSO as stock. Compound doses were obtained following dose response experiments and at the doses used no significant effect on cell viability was observed.

### High content imaging and analysis

The INCell Analyzer 2200 with high speed and autofocus fluorescent imaging was used to capture, segment and quantify the images. Protocols were optimised for segmenting Parkin puncta, mitochondria and differing cell types. A top hat algorithm was used to identify cell nuclei (Hoechst stain) and cell boundaries were defined using a multiscale top hat algorithm analysis of cell fluorescent signals.

### siRNA transfection

Transfections were carried out in 96-well plates after cells were seeded for 24 h. Lipofectamine RNAiMAX reagent (Fisher) and siRNAs were diluted in Opti-MED media (Fisher) to a final concentration of 1 pmol siRNA and final volume of 0.3 μL RNAiMAX,. An NTC smart pool (Thermo Fisher Scientific, D-001810-10-50) was used as a control, and a PINK1-targeting smart pool was used as a positive control (Thermo Fisher Scientific, L-004030-00-0050). ON-TARGETplus SMART pools for GSK3 and individual CDKs were purchased from Horizon Discovery. Human siRNAs targeting CDK1 (983) (L-003224-00-0005), CDK2 (1017) (L-003236-00-0005), CDK5 (1020) (L-003239-00-0005), CDK9 (1025) (L-003243-00-0005) and GSK3B (2932) (L-003010-00-0005) were used.

### MTT cell viability assay

Cell viability was measured by MTT assay. MTT powder was purchased from Sigma (M2128). Before experiments, MTT solution was premade to the stock centration of 5 mg/mL in PBS, filtered and kept at −20 °C before use. In each experiment, 10 μl media was taken out of the wells to test in a 96-well plate and replaced by 10 μl MTT stock solution to reach a working concentration of 0.5 mg/mL. Plates were then incubated for 2 h at 37 °C in an incubator before all the liquid was carefully removed and replaced by 100 μl freshly made solvent (Isopropanol: ethanol: DMSO: = 4:2:1). 10 min of gentle rocking at RT were required to dissolve the MTT formazan, after which the absorbance value was read at OD = 570 nm by a plate reader immediately. The actual value was corrected by deducting the value of wells without cells as the background. Cell viability values were then normalised to the control and was presented as fractions.

### Mitochondrial DNA analysis

Areal-time PCR-based mtDNA assay was used to measure mtDNA. DNA was extracted from cells and purified using a Wizard Genomic DNA Purification Kit (Promega), following the standard protocol for tissue culture cells. DNA concentration was measured using a nano-drop spectrophotometer (ThermoFisher Scientific). Samples were used immediately or stored at −20 °C and the qPCR was carried out using a StepOnePlusTM Real-Time PCR system (Thermo Fisher Scientific) using modified 2-h PCR protocols according to each primer's requirement. A mastermix was made with Power SYBR Green (ThermoFisher Scientific) following the standard recipe and the required pairs of primers were added into the plate for the qPCR process. Primers used for qPCR were

- RNA-Leu (UUR): CACCCAAGAACAGGGTTTGT/ TGGCCATGGGTATGTTGTTA.
- B2-microglobulin: TGCTGTCTCCATGTTTGATGTATCT/ TCTCTGCTCCCACCTCTAAGT

The relative mitochondrial DNA content to nuclear DNA was calculated using the following equations: Relative mtDNA content = 2 × 2^ΔCT^, ΔC_T_ = (b C_T_ –a C_T_).

### Seahorse experiments

Oxygen consumption rate (OCR) and extracellular acidification rate (ECAR) in live cells were measured using a Seahorse XFp analyser (Agilent) according to the manufacturer’s instructions. Cells were seeded into Seahorse XFp 96 well microplates at an optimised density and 24 h hours later, cells were washed and incubated with Seahorse XF Cell Energy Phenotype Assay Media.FCCP titration assays were performed to determine the optimum concentration for the Mito Stress Test use, which was performed using the Seahorse XFp Cell Mito Stress Test kit (Agilent). Cells were washed and kept with prepared assay media and incubated without CO_2_ for 45 min at 37℃. The standard Cell Mito Stress Test protocol was performed without further modification to allow OCR and ECAR to be calculated.

### Immunofluorescence staining

Cells were fixed and permeabilised with ice-cold methanol in a freezer for 20 min and simultaneously blocked with 10% normal goat serum (NGS) and 1% bovine serum albumin (BSA) for 1 h at room temperature. After blocking, cells were incubated with primary antibodies diluted in 10% NGS with 1% BSA PBS solution at 4 °C overnight. The cells were washed three times with PBS, and then secondary antibodies diluted in 10% NGS and 1% BSA PBS were applied for 1 h and covered to avoid light exposure. After incubation, samples were washed carefully with PBS (three times) Hoechst was used to stain nuclei at a dilution of 1:200.

### Western blotting

Cell lysis buffer (typically RIPA buffer) was used to lyse the cells on ice, and the protein supernatant collected following centrifugation. Protein concentrations were calculated using the Pierce BCA protein assay kit (Thermo Scientific)0.20 μg of heat denatured protein was loaded and run on pre-cast linear gradient gels (Bio-rad 161-1123EDU) A Trans-Blot Turbo transfer system (Bio-Rad) was used to transfer proteins to a PVDF membrane (Bio-rad 1704156EDU), which were blocked in 5% milk powder in PBS (w/v) for 1 h at RT and then probed with primary antibody diluted in 1% milk powder in PBS overnight at 4 °C. After washing with PBS-T, proteins were incubated with secondary antibody (peroxidase-linked anti-rabbit IgG (NA934, GE Healthcare) or anti-mouse IgG (NA931, GE Healthcare)) diluted in 1% skimmed milk powder for 1 h at RTEnhanced chemiluminescence (ECL) reagent was used to visualise the antibody-bound protein and G:Box F3 (Syngene) was used to image the light signal for the protein bands.

### Flow cytometry

Cells were harvested using Trypsin/EDTA, washed and centrifuged. Cells were resuspended in Zombie NIR (Biolegend) (pre-dissolved in DMSO, 1vial into 100μL) at 1:500 and then fixed with 4% PFA and stained with Hoechst. Samples (100μL in PBS) were then analysed a Novocyte Flow Cytometer. (20,000 single cells or 80% of samples were counted) The results were further analysed usingNoVo Express software. A traditional gating strategy based on forward and side scatter properties of the samples in the control (FSC and SSC) was applied to determine the cell population in each group for investigated. The square gate, ‘Live’, was set based on the (low) red intensity of the Zombie NIR™ dye staining (APC-Cy7-H) to discriminate the population from dead cells. The quadrant gate was set to sort apoptotic cells based on the high intensity of the Hoechst staining (Pacific Blue-H). All the gates were set in the control and applied unchanged on the other groups.

### SIRT1 assays

SIRT1 assays were carried out according to the manufacturer’s protocol (Wuhan Fine Biotech Co. Ltd.), H4 cells were lysed using RIPA lysis and extraction buffer mixed with protease and phosphatase inhibitor (1×). Protein was measured using Pierce™ BCA Protein Assay Kit (Thermo Scientific™ 23,225). The Human SIRT1 ELISA kit is based on sandwich enzyme-linked immunosorbent assay technology whereby the capture antibody was pre-coated onto 96-well plates and the biotin-conjugated antibody was used as detection antibodies. The same amount of cell lysate was loaded into the well for each treatment. The absorbance value was read at 450 nm by a plate reader immediately.

### Statistical analysis

Values were generally normalised to controls unless stated otherwise. Statistical analysis was carried out on experiments with a minimum of 3 biological replicates (n = 3). Statistical analyses were performed and presented using GraphPad Prism software including normalization. The student's t-test (unpaired, two-tailed) was used to compare two groups. ANOVAs with the suitable post-hoc tests were used for multiple groups. In all cases, p-values of < 0.05 were considered statistically significant. P-value significance levels when displayed in figures are denoted as follows: ns not significant, *p < 0.05, **p < 0.01, ***p < 0.001 and **** < 0.0001.

## Data availabiilty

The datasets used and/or analysed during the current study available from the corresponding author on reasonable request.

## Supplementary Figures

Supplementary Information.
