# Supplementary material for: AZD5438 a GSK-3a/b and CDK inhibitor is antiapoptotic modulates mitochondrial activity and protects human neurons from mitochondrial toxins
Source: Sci Rep. 2023 May 23;13:8334. doi: 10.1038/s41598-023-35480-2 (PMC10205901; doi:10.1038/s41598-023-35480-2)
Supplement: Supplementary file 1 — Supplementary Information. [file 41598_2023_35480_MOESM1_ESM.pdf]

AZD5438 a GSK-3a/b and CDK inhibitor is antiapoptotic modulates mitochondrial activity and protects human neurons from mitochondrial toxins

Running Title: AZD5438 protects neurons from mitotoxins

Gongyu Shi[1], Helen Scott[1], Nur Izzah Farhana Mohamad Azhar[1], Andriana Gialeli[1], Benjamin Clennell[1], Keng Siang Lee[1], Jenny Hurcombe[1,2], Daniel Whitcomb[1], Richard Coward[1,2], Liang-Fong Wong[1], Oscar Cordero-Llana[1], James B. Uney[*]

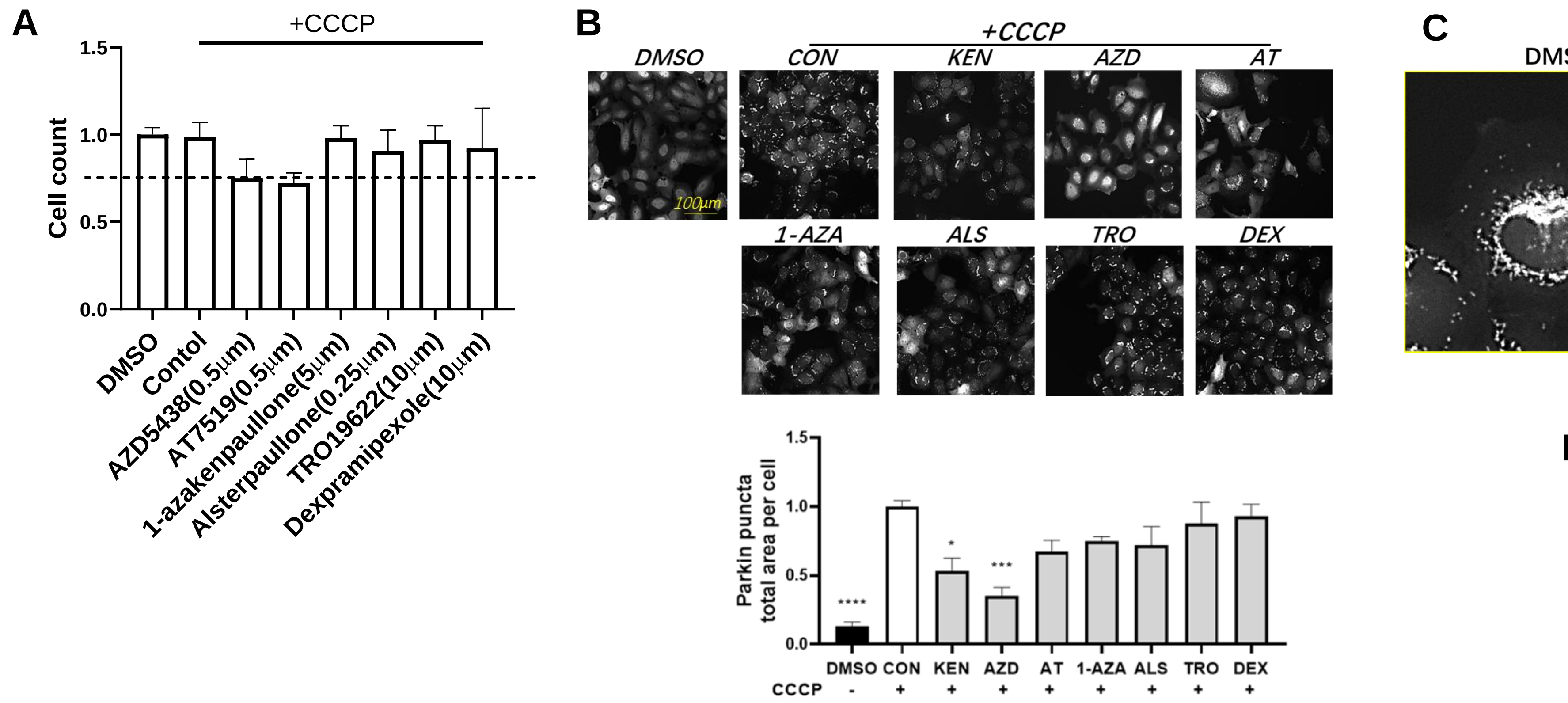

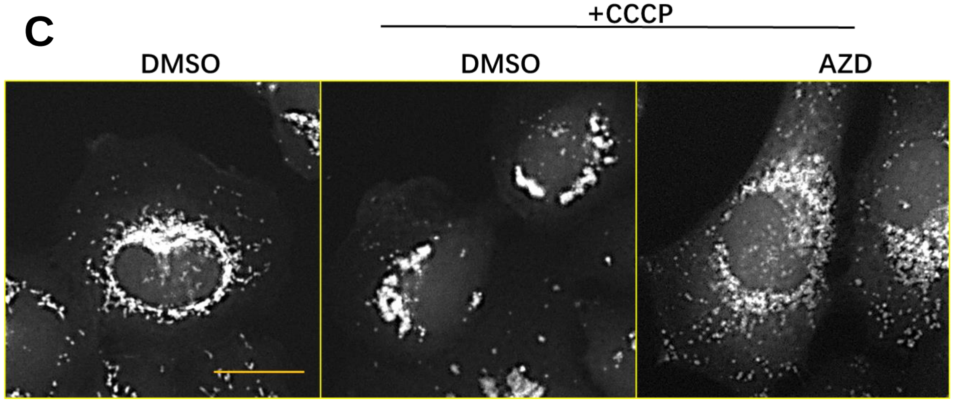

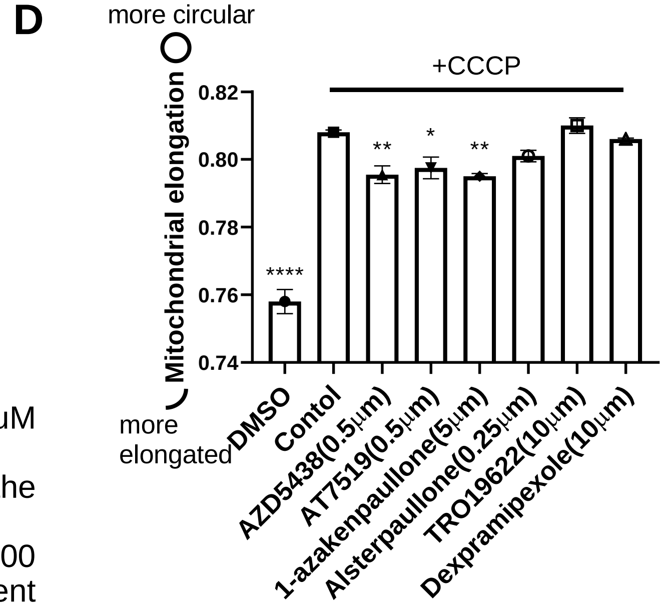

Supplemental Figure 1.

(A) H4-EGFP-PRKN cells were treated with the indicated GSK/CDK inhibitors for 24 hrs prior to incubation with CCCP (10uM for 2 hrs) and cell viability estimated by cell count (using the INCell Analyser 2200).
(B) The photomicrographs show representative images of EGFP-parkin recruitment in H4-EGFP-PRKN cells treated with the indicated GSK/CDK inhibitors for 24 hrs prior to incubation with CCCP (10uM for 2 hrs) to induce PRKN recruitment.
The bar graphs show values of the total area of the EGFP-parkin puncta per cell captured using an INCell Analyser 2200 (means + SEM of 3 independent experiments). A statistically significant increase in parkin recruitment is induced by treatment with CCCP (****p<0.0001) when compared to the DMSO control. AZD5438 and kenpaullone mediated a statistically significant inhibition of parkin recruitment (*** p<0.001 and *p<0.05 respectively).
(C) High magnification images from B (x20 objective images) of cells treated with CCCP and AZD5438 compared to DMSO controls as shown in B. Scale bar=25μm
(D) H4-EGFP-PRKN cells were treated with the indicated GSK/CDK inhibitors for 24 hrs prior to incubation with CCCP (10uM for 2 hrs) and mitochondrial morphology imaged using the INCell Analyser 2200. The results show that several of the GSK/CDK inhibitors prevented the increase in mitochondrial elongation seen following treatment with CCCP. Statistical analysis was carried out by one-way ANOVA test with Sidak's multiple comparisons. *p<0.05, ** p<0.01, *** p<0.001, **** p<0.000.1

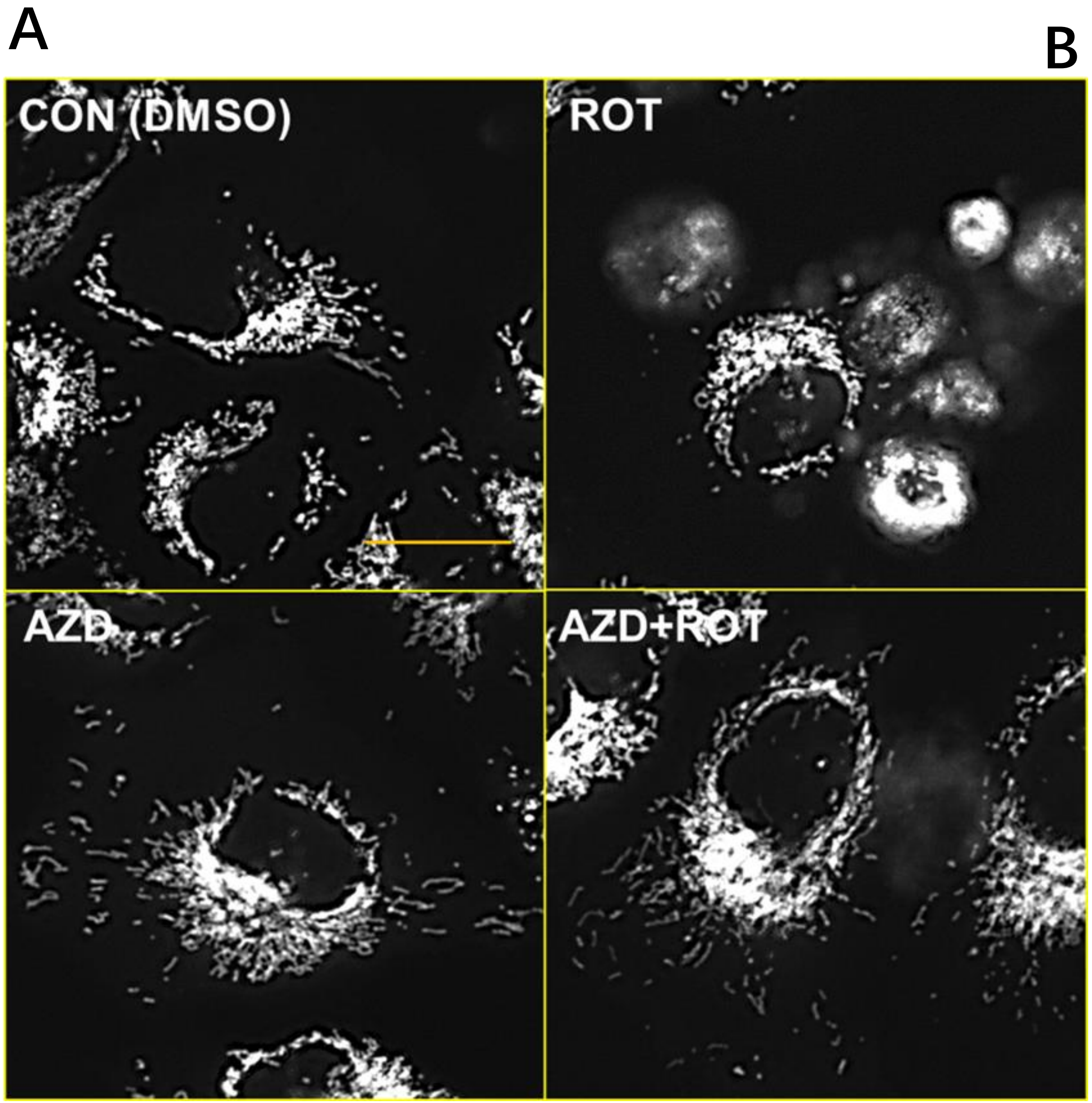

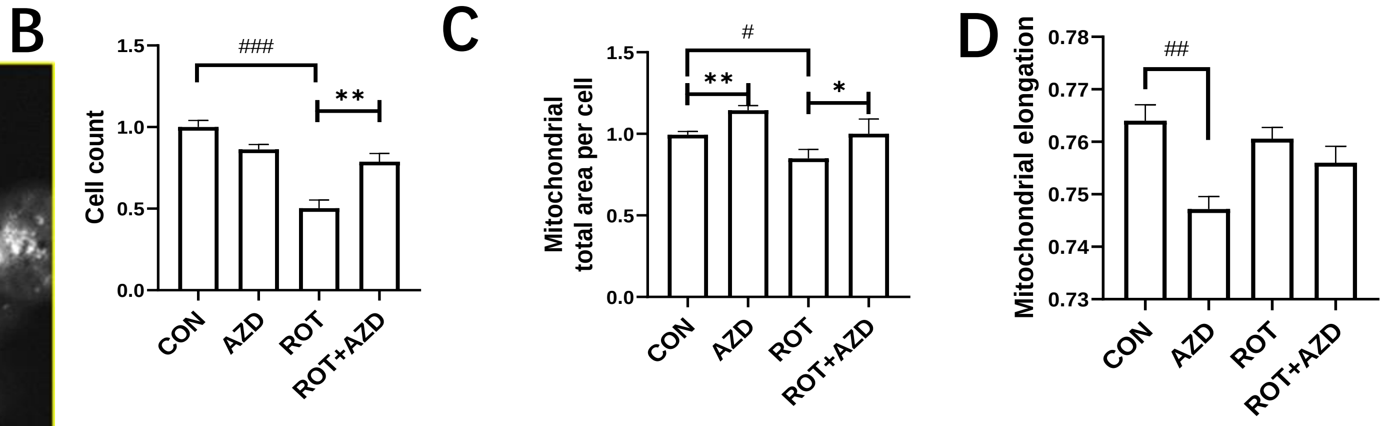

Supplemental Figure 2. Assessing the protective effect of AZD5438 on mitochondrial morphology and network following treatment with rotenone.

A) Representative high magnification images showing the Mitotracker stained mitochondrial network of cells treated with the indicated drugs. Arrow indicates the intense mitochondrial staining seen following treatment with ROT. Scale bar=25µm. B) Treatment with ROT significantly reduces the cell count (p=0.0002) while treatment with AZD prevents the loss in cell number (p= 0.0098). C) The total area of mitochondria per cell was significantly (p= 0.0058) increased when cells were treated with AZD compared to CON (DMSO vehicle). ROT treatment significantly (p= 0.0387) reduced the mitochondrial total area compared to CON. AZD mediated a significant protective effect (p= 0.0228) and prevented the decrease in mitochondrial associated with treatment with ROT.
D) Mitochondrial became more elongated following treatment with AZD (p= 0.0014). Statistical analysis was carried out by two-way ANOVA with Tukey's multiple comparisons test. N=3; data is displayed as mean ± SEM, significance is displayed as * p<0.05, ** p<0.01, **** p<0.0001. Asterisks denote comparisons between AZD and AZD+ROT treatment, hashes denote comparisons between CON and treatment groups.

Supplemental Figure 3. Unprocessed and uncropped western blots of caspase-3, PGC1-alpha and TOMM20.

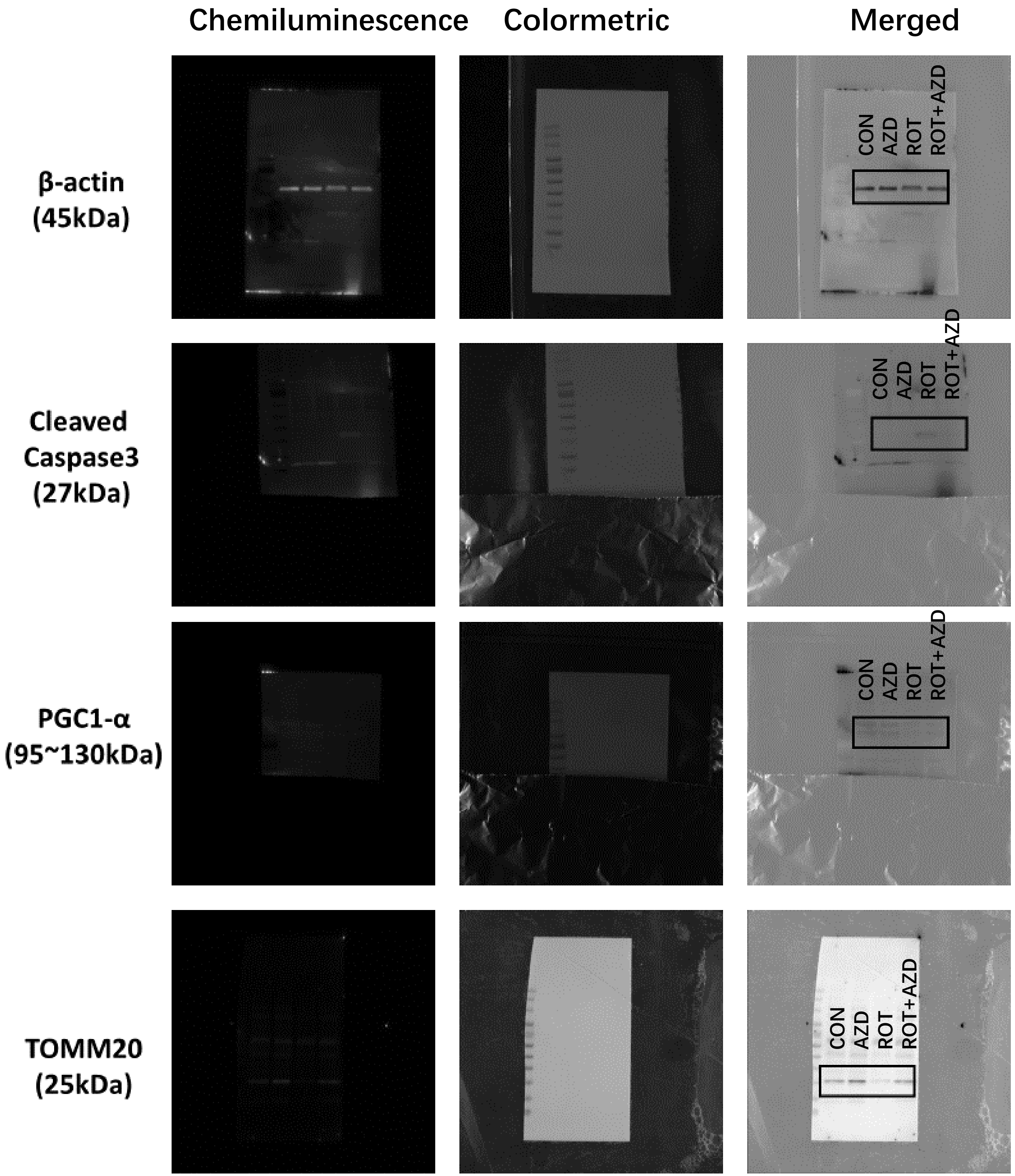

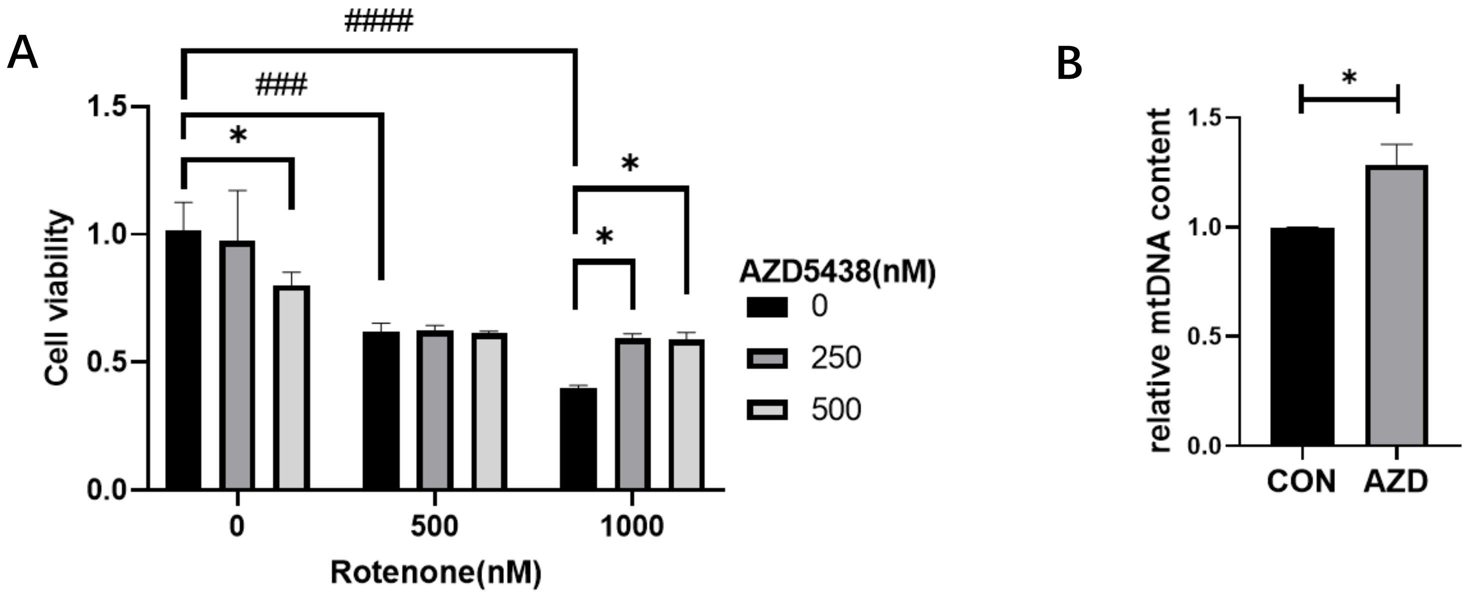

Supplemental Figure 4. AZD5438 protected SH-SY5Y cells from rotenone toxicity, and increased mtDNA levels.

A) SH-SY5Y-EGFP-PRKN cells were treated with rotenone and cell viability was assessed using MTT assays. Cells treated with AZD5438 were protected from the toxic effects of rotenone. B) AZD significantly increased the mtDNA content compared to CON ($p=0.0261$). Statistical analysis was carried out by two-way ANOVA test with Tukey's multiple comparisons test (A), unpaired t-test(B). N=3; data is displayed as mean ± SEM, significance is displayed as * $p<0.05$,

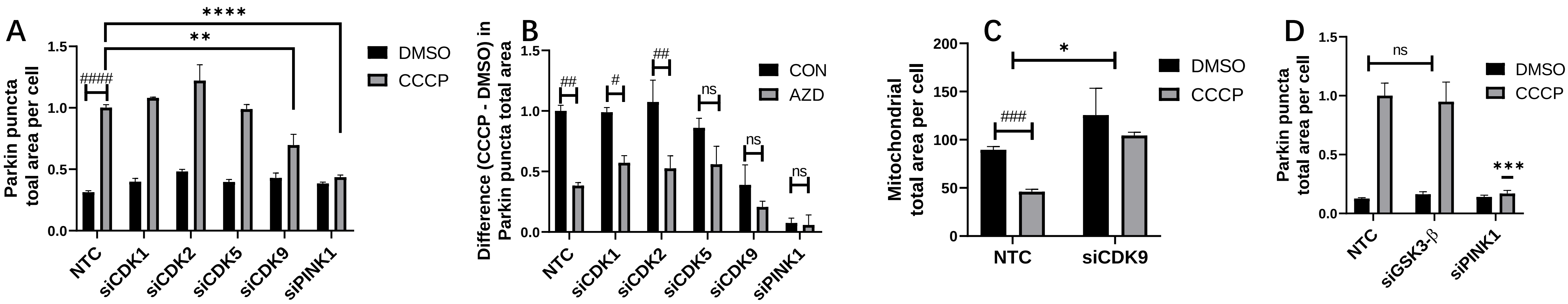

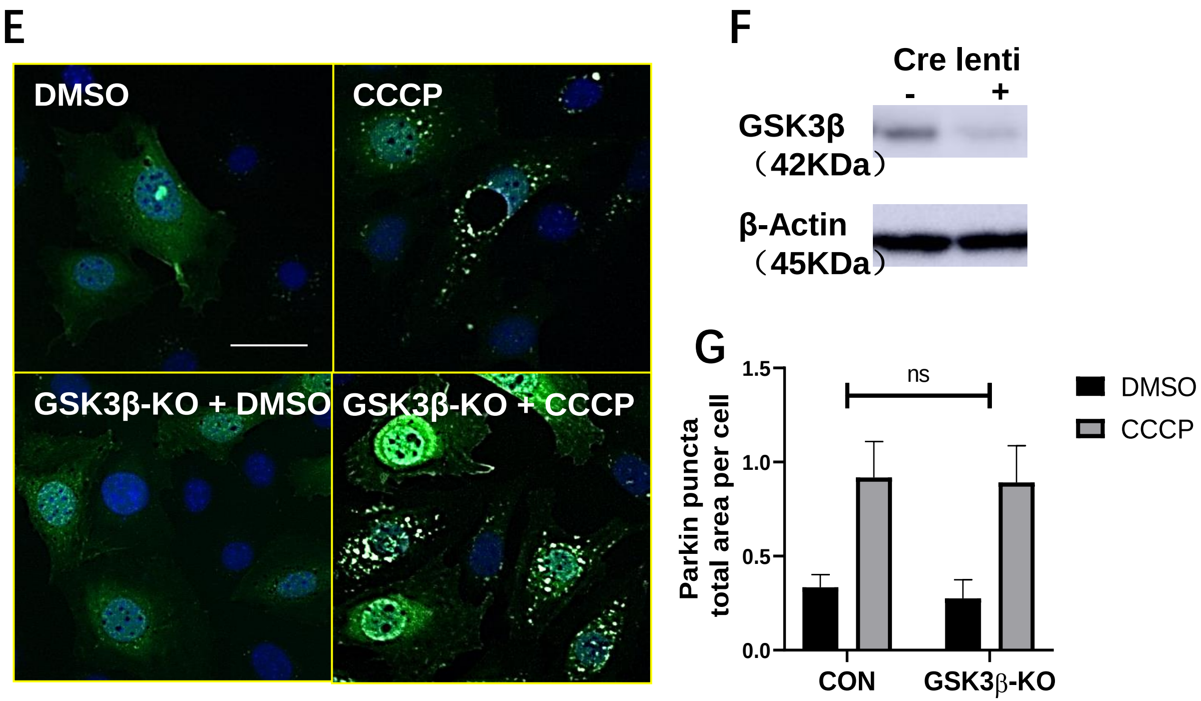

Supplemental Figure 5. Evaluating the actions of AZD5438 following the inhibition of CDKs and GSK-beta.

A) H4 cells stably expressing EGFP-parkin were treated with anti-CDK 1, 2, 5 and 9 siRNAs and EGFP-parkin puncta measured using the InCell analyser following treatment with CCCP.

(B) H4 cells stably expressing EGFP-parkin were transfected with anti-CDK siRNA 24hrs prior to treatment with AZD5438 (250nM) and EGFP-parkin puncta were measured following treatment with CCCP. The results show CCCP mediates an increase in Parkin recruitment and AZD5438 inhibits this in the presence of siRNAs against CDK1 and 2 but does not inhibit in the presence of anti- CDK5 and CDK9 siRNAs.

C) H4 cells were incubated with anti-CDK9 siRNA or a NTC and then exposed to 10uM CCCP. Mitochondria were labelled with MitoTracker Red and images captured.

(D) Transfection of an anti-GSK3-b siRNA into in H4-EGFP-PRKN did not alter parkin recruitment following treatment with CCCP.

E) The photomicrographs show EGFP-PRKN-podocyte cells following the lentiviral mediated cre-loxp mediated conditional knockdown of GSK3-b with/without the present of CCCP.

F) GSK3-b knockdown was confirmed using western blot following the transduction of the lentivirus expressing cre.

G) The bar graph shows GSK3-b knocking down in podocyte cells had no effects on parkin puncta total area (with/without CCCP). Statistical analysis was carried out by two-way ANOVA test with Tukey's multiple comparisons test (A, B, D, G). Statistical analyses was carried out using unpaired t-tests (C). N=3; data is displayed as mean ± SEM, significance is displayed as * $p<0.05$, ** $p<0.01$, *** $p<0.001$, **** $p<0.0001$

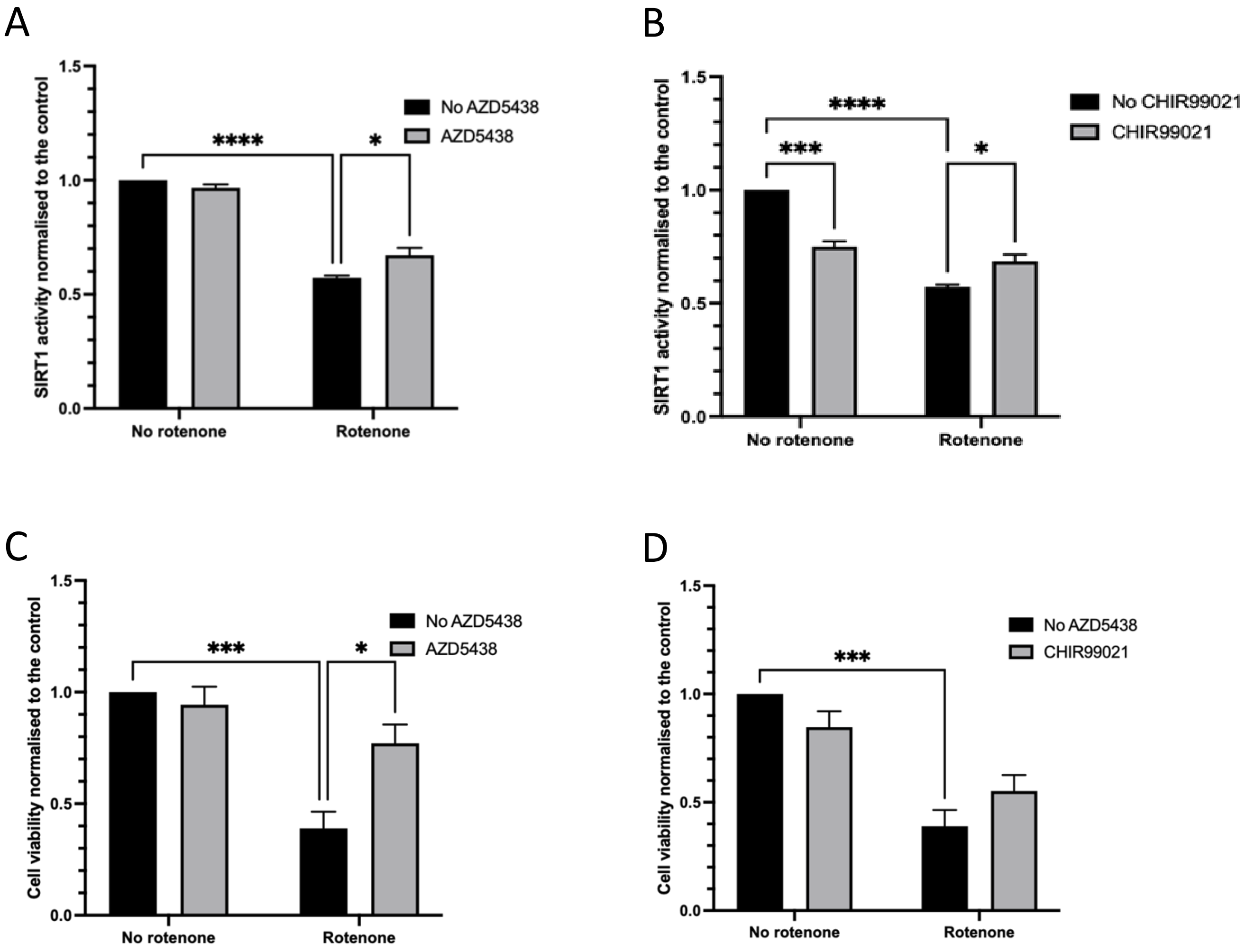

Supplementary Figure 6. The effect of AZD5438 and CHIR99021 on SIRT1 expression in rotenone challenged cells.

SIRT1 (A & B) expression and MTT activity (C &D) was measured in H4 cells in absence and presence of AZD5438 (GSK3 and CDK inhibitor) and of CHIR99021 (a GSK-3 specific inhibitor) following exposure to rotenone. The bar graphs in A & B show SIRT1 activity (means + SEM of 3 independent experiments). The bar graphs in C & D show cell viability (means + SEM of 4 independent experiments) as assessed by MTT assay.
Statistical analysis was carried out by Two-way ANOVA test with Sidak's multiple comparisons. *p<0.05, **p<0.01, ***p<0.001, ***P<0.0001

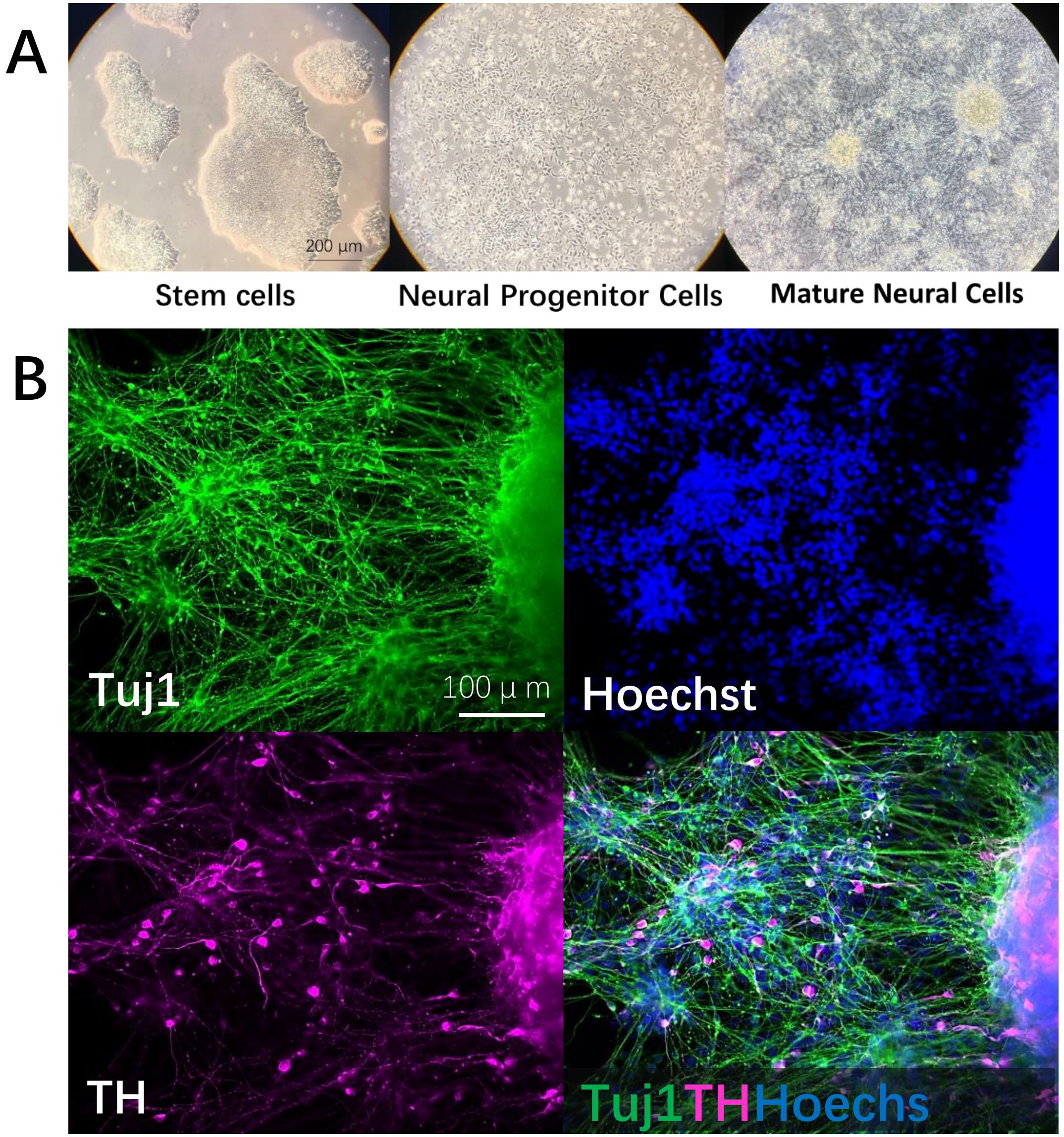

C

Percentage TUJ1+/HOESCHT	Percentage TH/TUJ1
33.5±7.5	25.4±10.3

Supplemental Figure 7. Midbrain neuronal cell culture generated from hiPSCs

A) Brightfield images showing the morphology of the cells at specific stages of the differentiation process. Scale bars =200 µm. B) Images NAS2 iPSCs that have been differentiated into midbrain dopaminergic neurons at day 45. Midbrain neuronal cell culture differentiation was characterised by visualising the expression of both the neuronal marker TUJ1 and the dopaminergic neuronal marker TH. C) Table shows the average percentage (±SD) of cells expressing TUJ1 and the percentage which express TH (n=3), analysed via ImageJ.

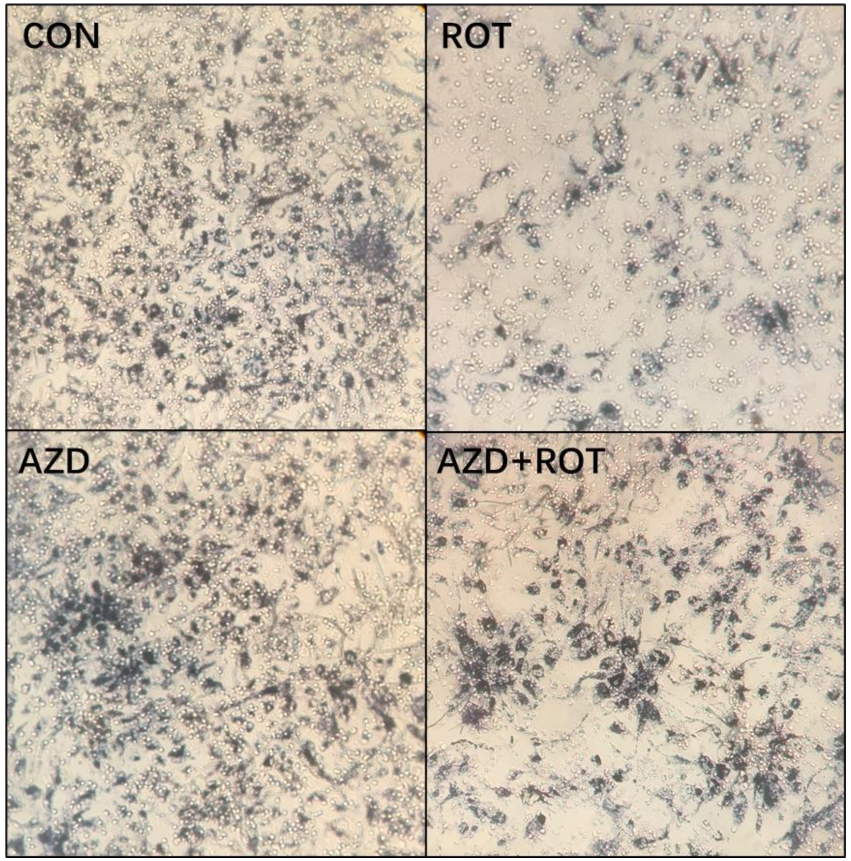

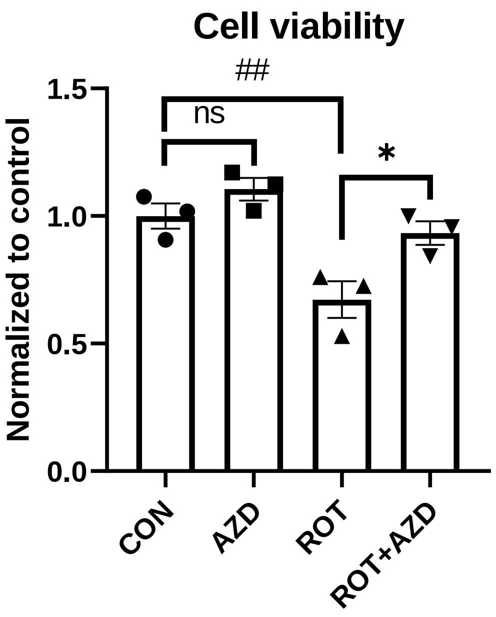

Supplemental Figure 8. AZD5438 protects human midbrain cells against rotenone-induced damage

A) Bright-field images show MTT formazan formation (dark staining) in each of the treatment groups (500nM AZD5438, 500nM rotenone, 500nM AZD5438+500nM rotenone). B) The bar graph shows the reduction in cell viability (as assessed by MTT assay) of the midbrain neuron cultures induced by rotenone was prevented by AZD5438 treatment. Statistical analysis was carried out by two-way ANOVA test with Tukey's multiple comparisons test. n=3 plates, data is displayed as mean ± SEM. *p<0.05, ** p<0.01.

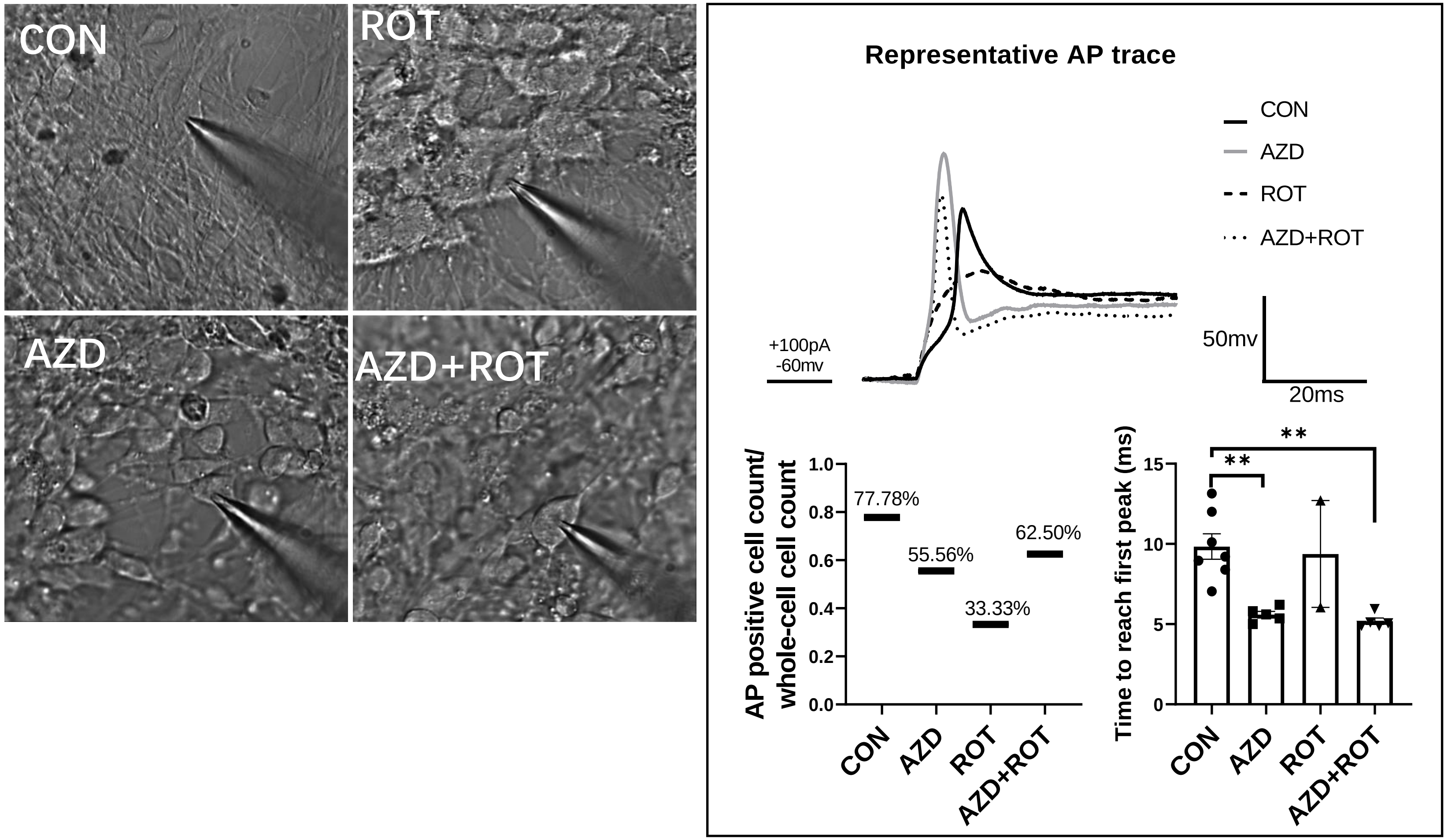

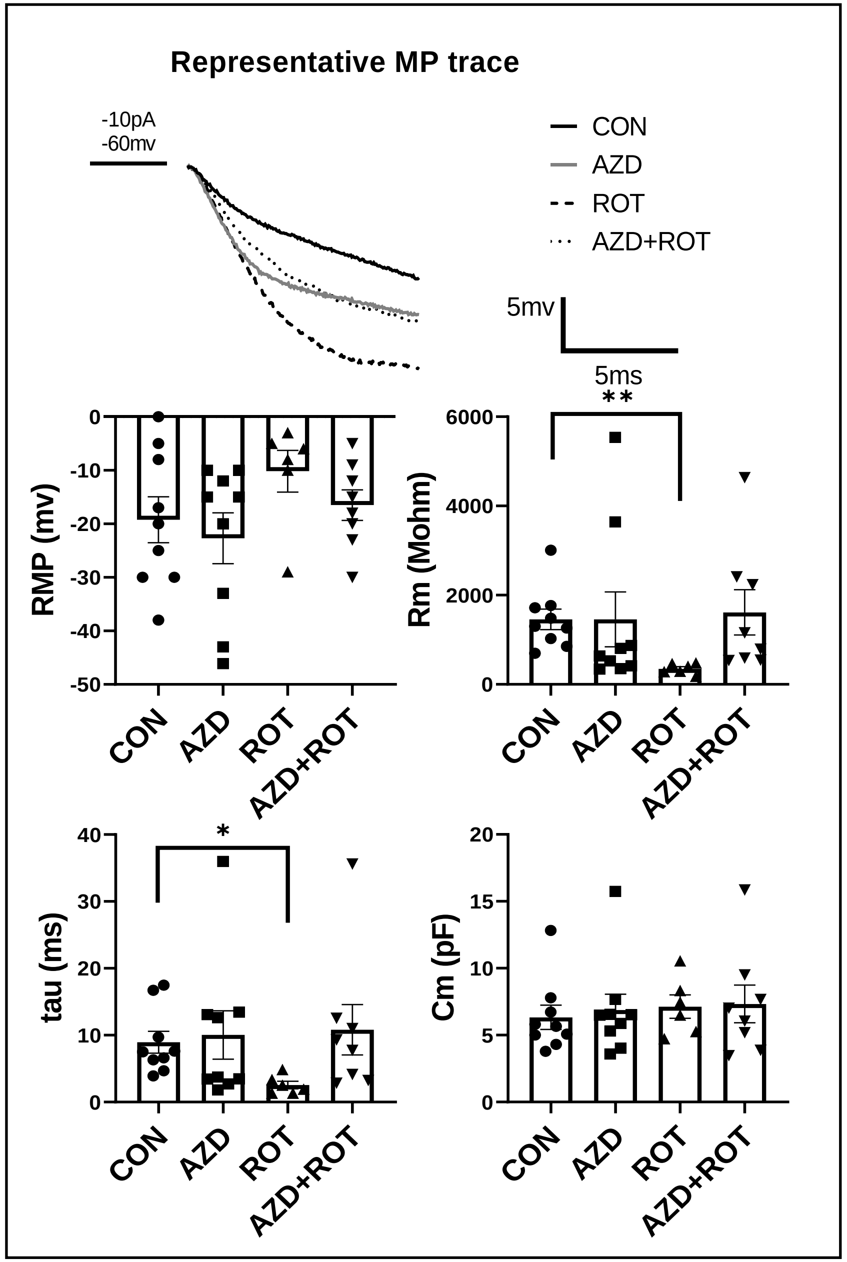

Supplemental Figure 9. AZD5438 prevents the loss of membrane resistance in human midbrain derived neuronal cultures associated with rotenone treatment

Brightfield photomicrographs of patch-clamped midbrain neurons derived from human iPSCs. Graphs show a representative trace of an action potential (AP) when cells were held at -60mv with an injection of +100pA, action potential positive cell ratios and the time to reach first peak were calculated from the AP data. Statistical analysis was carried out by Welch's ANOVA test with Dunnett's T3 multiple comparisons test. Graphs also show the representative traces of membrane potential (MP) recorded at -60mv with an injection of -10pA. The resting membrane potential (RMP), membrane resistance (Rm), membrane capacity (Cm) and time constant (tau) calculated from the MP data. C)For MP data, N=9(CON),9 (AZD),6(ROT) and 8 (AZD+ROT); for AP data, N=7(CON),5(AZD),2 (ROT) and 5 (AZD+ROT), data is displayed as mean ± SEM. *$p<0.05$, ** $p<0.01$.
